# Effectiveness of telemedicine-delivered exercise interventions in older adult patients with osteoarthritis: a systematic review and meta-analysis

**DOI:** 10.3389/fpubh.2025.1719841

**Published:** 2025-12-03

**Authors:** Jing Zhu, Yue-shuai Jiang, Hexia Wang

**Affiliations:** School of Education, China University of Petroleum, Beijing, China

**Keywords:** osteoarthritis, meta-analysis, older adult, telemedicine, systematic review

## Abstract

**Background:**

Remote rehabilitation treatment delivered via the Internet may serve as an effective approach for managing osteoarthritis (OA). One of its primary advantages is that patients can conveniently access rehabilitation services at any time and from any location. However, there remains a lack of reliable and up-to-date systematic reviews and meta-analyses to confirm the effectiveness of this therapy in improving clinical outcomes for older adult with OA.

**Purpose:**

Our study aims to conduct a systematic review and meta-analysis of the available evidence regarding the effectiveness of telerehabilitation in older adult patients with OA.

**Methods:**

A systematic search was performed in the Medline, Web of Science, Cochrane databases, and Embase from their inception up to August 2025. Trials focusing on the effects of telerehabilitation for older adult individuals suffering from OA were included. Two reviewers, working independently, carried out the selection of studies and the extraction of data. The assessment of bias risk utilized the Cochrane Risk of Bias tool.

**Results:**

We selected 13 randomized clinical trials, which included a total of 1,845 participants. Overall quality of the included studies was rated as moderate-to-high quality. Our findings indicate that telerehabilitation effectively alleviates pain (standardized mean difference, SMD: 0.40, 95% confidence interval, CI: 0.11 to 0.69, *I*^2^ = 88.3%), enhances physical function (SMD: 0.61, 95% CI: 0.16 to 1.05, *I*^2^ = 91.5%), and improves the quality of life (SMD: 0.42, 95% CI: 0.07 to 0.77, *I*^2^ = 90.1%) in older adult individuals suffering from OA. The subgroup analysis results showed that remote exercise interventions based on APP, mobile phones, and web platforms were all effective in improving the pain levels of older adult patients with OA, and interventions with a duration of more than 3 months significantly enhanced the improvement in pain.

**Conclusion:**

Remote exercise rehabilitation interventions have been shown to effectively alleviate pain, improve quality of life, and enhance physical function in older adult individuals with OA. This approach offers a viable alternative to traditional face-to-face exercise rehabilitation for older adult patients suffering from OA.

**Systematic review registration:**

PROSPERO: CRD420251159586.

## Introduction

Osteoarthritis (OA) is the most prevalent form of arthritis and is classified as a chronic degenerative musculoskeletal disorder. The pathophysiological process of OA involves several critical stages, including cartilage degeneration, synovial inflammatory responses, bone hyperplasia, osteophyte formation, and subchondral bone remodeling (1). Clinically, OA is characterized by joint pain, stiffness, swelling, morphological deformities, and motor dysfunction, which significantly impair patients’ daily activities (2). Epidemiological studies estimating that approximately 250 million individuals worldwide suffer from osteoarthritis (3). As age increases, obesity rates rise, and life expectancy extends, the incidence of OA continues to escalate. About 10% of individuals aged 60 and above exhibit typical symptoms, with prevalence further increasing to 40% among those over 70 (4), rendering OA a significant contributor to the decline in motor function among older adult individuals. The 2019 Global Burden of Musculoskeletal Diseases survey indicated that osteoarthritis accounts for 20.1% of musculoskeletal rehabilitation needs, imposing a substantial economic and public health burden on individuals, families, and society (5), thereby underscoring the urgent need for effective treatment interventions.

The primary objectives of clinical treatment for arthritis are to alleviate pain, slow disease progression, and enhance joint function (6). Current treatment strategies integrate exercise interventions with surgery, medication, and other modalities (7). Existing studies have demonstrated that exercise can improve physical function, enhance quality of life, and alleviate pain in OA patients (8, 9). Additionally, for patients with early mild OA, participation in self-management programs can effectively relieve clinically significant pain (10). However, due to limitations in resource access, time, and cost, many patients struggle to consistently engage in long-term follow-up and rehabilitation, which significantly diminishes their recovery outcomes. Therefore, it is imperative to explore new intervention strategies to provide effective rehabilitation for patients with knee osteoarthritis.

The core definition of mobile health is the provision of medical services and public health practices conducted through mobile devices such as smartphones, portable patient monitors, personal digital assistants, and other wireless communication devices (11). Numerous high-quality review studies have confirmed that exercise rehabilitation interventions based on mHealth technology are effective in improving pain levels, joint dysfunction, and other associated symptoms in patients with OA (6, 12, 13). During the COVID-19 pandemic, the forced suspension of non-urgent orthopedic surgeries resulted in a significant increase in the demand for remote rehabilitation services among OA patients. Utilizing its remote delivery capabilities, remote rehabilitation intervention quickly emerged as an effective alternative for pain management in OA patients.

Current studies have confirmed that mHealth exercise interventions achieve efficacy equivalent to traditional face-to-face clinical interventions and can be implemented under strict adherence to medical safety standards (14). For older adult patients with OA, despite facing challenges with internet operation skills, their limited mobility and poor spatial movement capabilities create a genuine need for remote rehabilitation interventions. Current research has demonstrated that remote rehabilitation interventions can effectively alleviate pain and improve the quality of life for young and middle-aged arthritis patients (12, 15). However, the effectiveness of these interventions for older adult individuals remains unknown. Meanwhile, existing meta-analyses focusing on older adult populations have often incorporated studies with mixed age groups, specifically those with a mean age of 60 years or older. This practice further exacerbates the heterogeneity of the findings and diminishes the applicability and generalizability of the results. Therefore, this study aims to explore the rehabilitation effects of remote rehabilitation interventions for older adult patients with OA in comparison to conventional interventions through a meta-analysis.

## Methods

The implementation of this meta-analysis strictly adhered to two core guidelines: the first being the relevant recommendations proposed in the Cochrane Handbook (16), and the second being the PRISMA extension statement, which is specifically applicable to systematic review studies that include pairwise meta-analyses (17). Since all analytical processes in this study were based on previously published research findings, without involving new human trials or the collection of original data, it did not require approval from an ethics committee, nor was it necessary to obtain informed consent from patients. This study has been registered on PROSPERO (CRD420251159586).

## Study selection and search strategies

All references were organized using Endnote X9 software (Thompson ISI Research Soft, Philadelphia, PA), and any duplicate or overlapping publications were automatically eliminated. Two independent authors assessed the titles and abstracts from the initial search, and any differences during this process were resolved through discussion or adjudicated by a third author. Following this, an additional thorough evaluation of the full texts was conducted to verify the accuracy and integrity of the studies.

A comprehensive strategic search of the literature was conducted to identify relevant randomized controlled trials (RCTs) concerning the effectiveness of mHealth-based exercise interventions for older adult individuals with OA from the following databases: Web of Science, PubMed, Embase, and Cochrane, covering their inception up to August 10, 2025. The studies were screened by employing Boolean logic operators alongside medical subject headings and relevant keywords in English. The terms used, either individually or in combination, included: “telemedicine,” “osteoarthritis,” “sports intervention,” “older adult,” and “RCT.” A series of manual recursive searches were conducted to complement the retrieval from top-tier journals (such as Osteoarthritis and Cartilage or the British Journal of Sports Medicine) and significant international conference proceedings to ensure that relevant articles that met our inclusion criteria (18, 19) were not overlooked. Additionally, manual searches were performed on the references of reviews regarding telemedicine-delivered exercise interventions as well as articles presented in abstract form. The specific search strategies utilized across all databases are detailed in Supplementary Appendix 1.

### Inclusion criteria

This study adopted the following inclusion criteria: (1) Study participants: study participants included older adult individuals aged 60 and above who have been diagnosed with OA. This demographic is particularly noteworthy, as it exhibits a higher prevalence of OA compared to younger and middle-aged populations, making them more susceptible to the adverse effects of arthritis (4, 20); (2) Interventions: any telemedicine-based exercise intervention programs were eligible, with no restrictions on exercise duration, cycle, content, or load; (3) Control group: the control group could consist of either routine interventions or a blank control; (4) Outcome measures: the primary outcome measure was pain, assessed using valid and reliable scales, while the secondary outcome measures included physical function and quality of life. When multiple scales were employed to assess a single outcome measure in an individual study, the primary measurement for that outcome was adopted. If a study did not specify the primary outcome measure, the most commonly used assessment scale for that outcome was utilized; (5) Study design: only English-language publications were included to ensure consistency.

### Data extraction and quality assessment

From the studies that met the inclusion criteria, we extracted several key data points, including author names, sample size, age, duration of intervention, gender ratio, intervention methods, outcome measures, and geographical region. In instances where essential data were not reported in the literature, we proactively reached out to the corresponding authors to obtain the necessary information.

Two independent evaluators conducted a risk assessment of each study using the Cochrane bias analysis framework. In instances of discrepancies in data collection and methodological judgments, a third-party reviewer intervened to mediate, ultimately facilitating a consensus through consultation (21). This evaluation system comprises seven core elements, categorizing the included studies as having uncertain risk, low risk, or high risk based on the following criteria: randomization procedure implementation, allocation concealment, blinding of participants and researchers, blinding of outcome assessment, data completeness, selective outcome reporting, and other potential biases.

The Grading of Recommendations Assessment, Development and Evaluation (GRADE) system was employed to evaluate the evidence quality of each statistically significant outcome measure. GRADE categorizes evidence quality into four tiers: high, moderate, low, and very low. The evidence grade may be downgraded by one level owing to several factors, such as risk of bias, inconsistency, indirectness of evidence, imprecision, and publication bias. Conversely, the evidence grade can be upgraded by one level if there is a significant effect size, a dose–response relationship, or if all potential biases only diminish the manifestation of treatment effects (22).

### Statistical analyses

In accordance with the guidelines of the Cochrane Collaboration, this study performed a traditional meta-analysis utilizing the random-effects model via STATA version 14.0 statistical software (Stata, Inc., College Station, TX) (23). Heterogeneity was quantified using the *I*^2^ statistic, with thresholds of 25, 50, and 75% representing low, moderate, and high levels of heterogeneity, respectively. Additionally, a Q-test result with a *p*-value below 0.1 indicated statistically significant heterogeneity (24). For continuous variables, the study calculated the standardized mean difference (SMD, defined as the ratio of mean difference to standard deviation), along with the 95% confidence interval (CI). To identify potential publication bias, a comparison-adjusted funnel plot was constructed, allowing for preliminary judgment based on the graph’s symmetry, supplemented by Egger’s test for quantitative verification, with a *p*-value threshold set at 0.05 (25). Furthermore, to explore heterogeneity and statistical significance among trials, a systematic subgroup analysis was conducted based on variables such as intervention duration, sample size, outcome measurement indicators, geographical distribution, and intervention methods (including telephone, APP, or websites).

## Results

### Literature selection and characteristics of included studies

**Figure 1:**
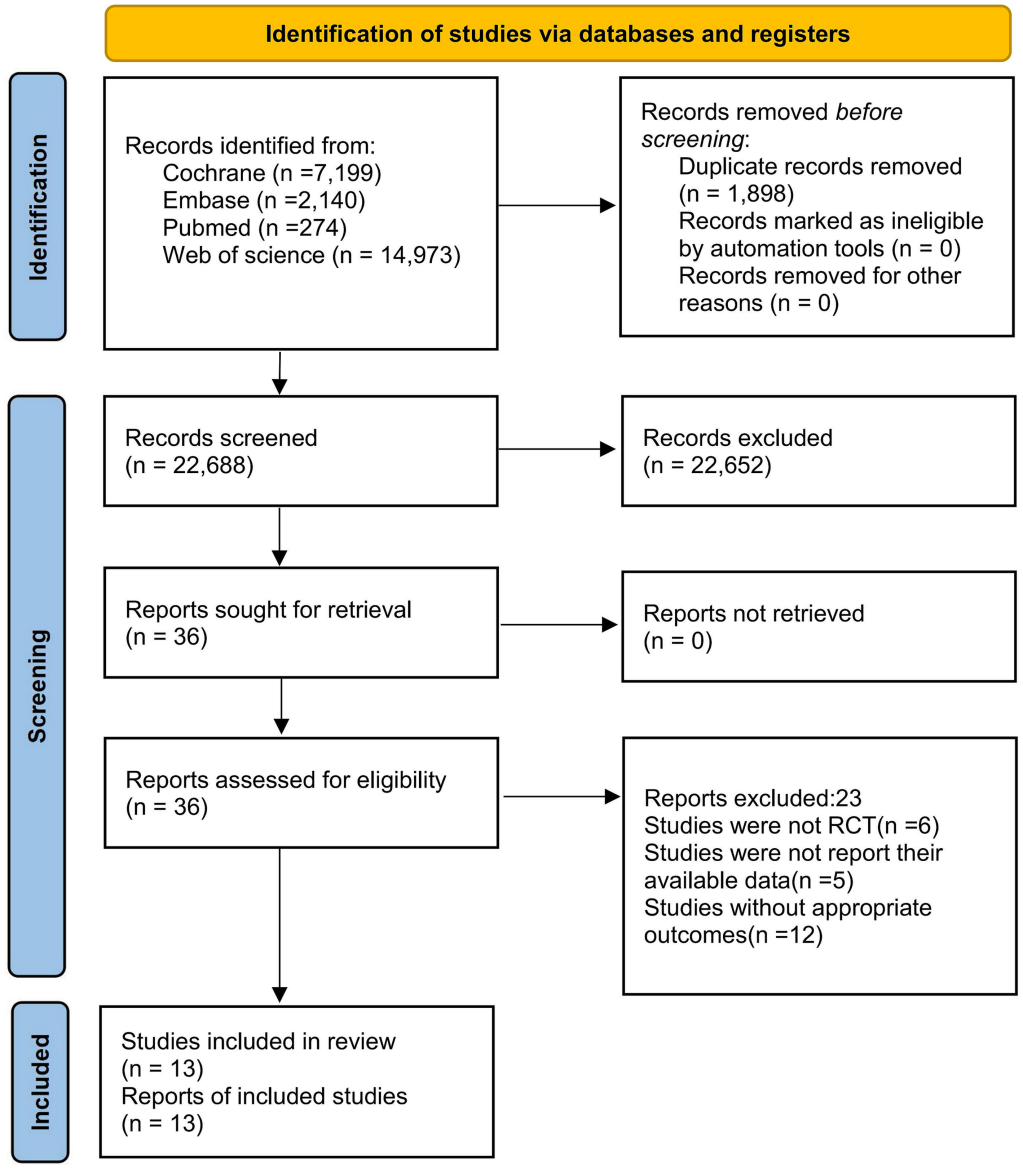
Literature review flowchart. WOS, web of science.

An initial literature search yielded 24,586 records. After the removal of 1,898 duplicate entries, 22,688 articles proceeded to the title and abstract screening phase, with only 36 articles advancing to further evaluation. Ultimately, 13 papers met the established research criteria (26–38). To ensure that potentially relevant studies were not overlooked, we also reviewed the reference lists of similar meta-analyses and reviews. The article selection process is illustrated in the PRISMA flow diagram presented in Figure 1. The search retrieved 13 trials published between 2016 and 2025. The telerehabilitation intervention group comprised 932 participants, while the control group included 913 participants. All study subjects were required to meet the criteria of having an average age over 60 and being diagnosed with OA. The intervention period varied from 4 weeks to 2 years. Table 1 summarizes the characteristics of the included studies and their participants.

**Table 1:** Demographic characteristics of included studies.

Publication	Sample size		Age, mean (SD)		Female (%)		Intervention		Intervention format	Intervention duration	Outcome(s)	Country
	EG	CG	EG	CG	EG	CG	EG	CG				
Zhu et al. (30)	32	28	68.59 ± 7.16	67.64 ± 7.85	59.38	53.57	Participants in the intervention group received interventions via a computer vision-based exercise assessment and intervention system, which conducted comprehensive knee function assessments, provided precise exercise interventions and real-time feedback, issued timed tasks.	Exercise education brochure	APP	6 weeks	ASES-8, GDS, Pain intensity, Physical function, QoL, ROM, UEQ, Stiffness	China
Li et al. (28)	25	26	64.8 ± 9	65.0 ± 8	76.00	88.46	Group education, a Fitbit, access to FitViz, and 4 biweekly phone calls from a PT over 8 weeks. Participants then continued using Fitbit and FitViz independently up to week 12.	Delay group	APP	12 weeks	Pain intensity, Physical function, MVPA time, ADL, QoL, Self-management, Motivation	Canada
Lee et al. (26)	15	16	65.63 ± 3.7	68.27 ± 4.8	100.00	100.00	Participants perform exercises at home using the app to control movement and repetitions precisely, along with the exoRehab remote rehabilitation device.	No intervention	APP	8 weeks	Pain intensity, Muscle biomarkers	Korea
Thiengwittayaporn et al. (27)	42	40	62.2 ± 6.8	63.0 ± 9.7	85.71	92.50	Patients received instruction of exercise via “Love your knee” mobile app.	Handout exercise guidance	APP	4 weeks	Pain intensity, Physical function, ROM, Recreation activities, QoL	Germany
Dieter et al. (29)	30	31	61.5 ± 7.5	64.2 ± 9.3	40.00	58.06	App-based exercise training.	Usual care	APP	12 weeks	Pain intensity, Physical function, QoL, Exercise-specific Self-efficacy, Isometric maximum force measurement, Postural control	Germany
Kloek et al. (31)	109	99	63.8 ± 8.5	62.3 ± 8.9	32.10	32.30	The e-Exercise group received approximately 5 face-to-face physical therapy sessions and an online application containing graded activity, exercise, and information modules.	The control group received exercise rehabilitation education videos with instructions for daily exercise movements similar to those in the intervention group’s application, without using the computer vision-based application.	APP	3 months	Physical function, Pain intensity, QoL, Self-Efficacy, Physical Activity, Treatment Engagement, Usability	Netherlands
Pelle et al. (33)	214	213	62.1 ± 7.7	62.1 ± 7.0	45.58	33.96	Using the dr. Bart app based on the tiny habits method, which includes an exercise library with 10 important exercises for the treatment of hip/knee osteoarthritis.	Usual care	APP	3 weeks	Number of secondary health care, Health care utilization, consultations, Physical function, Pain intensity, QoL, Physical Activity, Illness perceptions	Netherlands
Bennell et al. (34)	84	84	64.8 ± 9	65.0 ± 8	211.11	140.00	5×30 minute consultations with a physical therapist, and 6–12 telephone coaching sessions by clinicians.	Routine consultations	Telephone	6 weeks	Physical function, Pain intensity, QoL, Physical Activity, Exercise adherence	Australia
Hinman et al. (35)	87	88	62.4 ± 9.1	62.5 ± 8.1	58.18	60.00	Exercise advice and support (5–10 consultations with a physiotherapist trained in behavior change for a personalised strengthening and physical activity programme) plus telephone service.	Telephone service	Telephone	6 weeks	Physical function, Pain intensity, QoL, Physical Activity, Satisfaction with care, Cost-effectiveness, Adverse events	Australia
Nelligan et al. (36)	103	103	60.3 ± 8.2	59.0 ± 8.5	71.67	56.06	Access to a website with educational information on osteoarthritis, a 24-week self-directed strengthening exercise program, and guidance on physical activity, and receipt of automated behavior-change text messages to encourage exercise adherence.	Only had access to a custom-built website containing information on osteoarthritis and general recommendations on the importance of exercise and physical activity.	Telephone	24 weeks	Pain intensity, Physical function, QoL, Overall treatment satisfaction, Adverse events, Cointervention use, Exercise adherence	Australia
Baker et al. (37)	52	52	64.5 ± 8.3	65.8 ± 6.6	23.81	23.81	Experimental group, post 6-week group strength training, had 24-month phone intervention (weekly motivational counseling first 6 months, then monthly) plus monthly auto reminder calls and booster sessions as needed.	Control group, post 6-week group strength training, only had 24-month monthly auto reminder calls and booster sessions as needed.	Telephone	2 years	Exercise adherence, Muscle strength, Number of telephone-linked communication calls received, Physical function, Pain intensity	United States
Bennell et al. (38)	107	105	62.8 ± 8.2	61.8 ± 7.2	52.86	34.62	Receive consultations with a physiotherapist over 6 months, and simultaneously receive 6–12 telephone coaching sessions on exercise and physical activity provided.	Receive consultations with a physiotherapist over 6 months.	Website	12 week	Adverse events, Balance confidence Depression, Anxiety, Stress, Exercise adherence, Fear of movement, Knee stiffness, Physical function, Pain intensity, QoL	Australia
Weber et al. (32)	32	28	60 ± 6	64 ± 8	34.40	42.90	A 12-week Join2Move app-based intervention, including graded activity, exercise, and education modules, with no physical therapy for the affected joint received during the study period.	Receive usual care without using the Join2Move app.	APP	12 weeks	Usability, Pain intensity, Physical Functioning, Physical Performance, Physical Activity, Self-management, User Satisfaction	Germany

ADL, Activities of daily living; CG, Control group; EG, Experimental group; FitViz, a Fitbit-compatible app; Geriatric Depression Scale, GDS; MVPA, moderate-to-vigorous physical activity; PT, physiotherapist; QoL, Quality of life; ROM, Range of motion; Arthritis Self-Efficacy Scale, ASES-8; User Experience Questionnaire, UEQ.

### Quality of included studies

The quality of individual studies and the overall study quality are illustrated in Supplementary Figures 1, 2, respectively. All 13 trials demonstrated adequate random sequence generation, with 6 randomized controlled trials revealing their methods of allocation concealment. While all randomized controlled trials exhibited uncertainties regarding performance bias, 1 trial was identified as having a high risk of performance bias, and 5 trials were found to have a high risk of detection bias. Furthermore, 9 studies indicated a low risk of attrition bias. Among the remaining bias categories, 7 trials were assessed as having a low risk of bias.

## Primary outcome

### Effect of telemedicine-delivered exercise on pain in older adult individuals with OA

**Figure 2:**
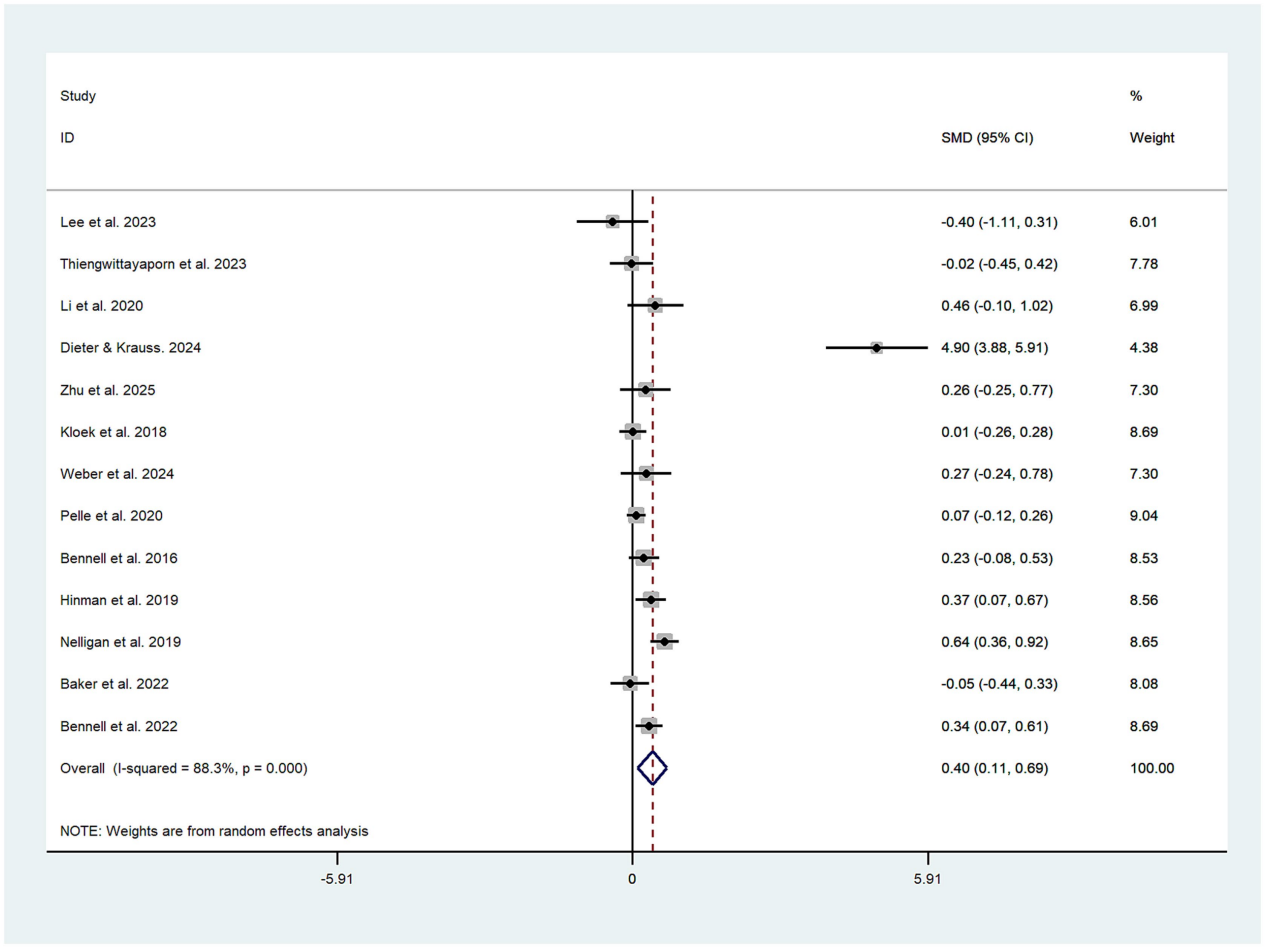
Literature review forest plot based on primary outcome.

**Figure 3:**
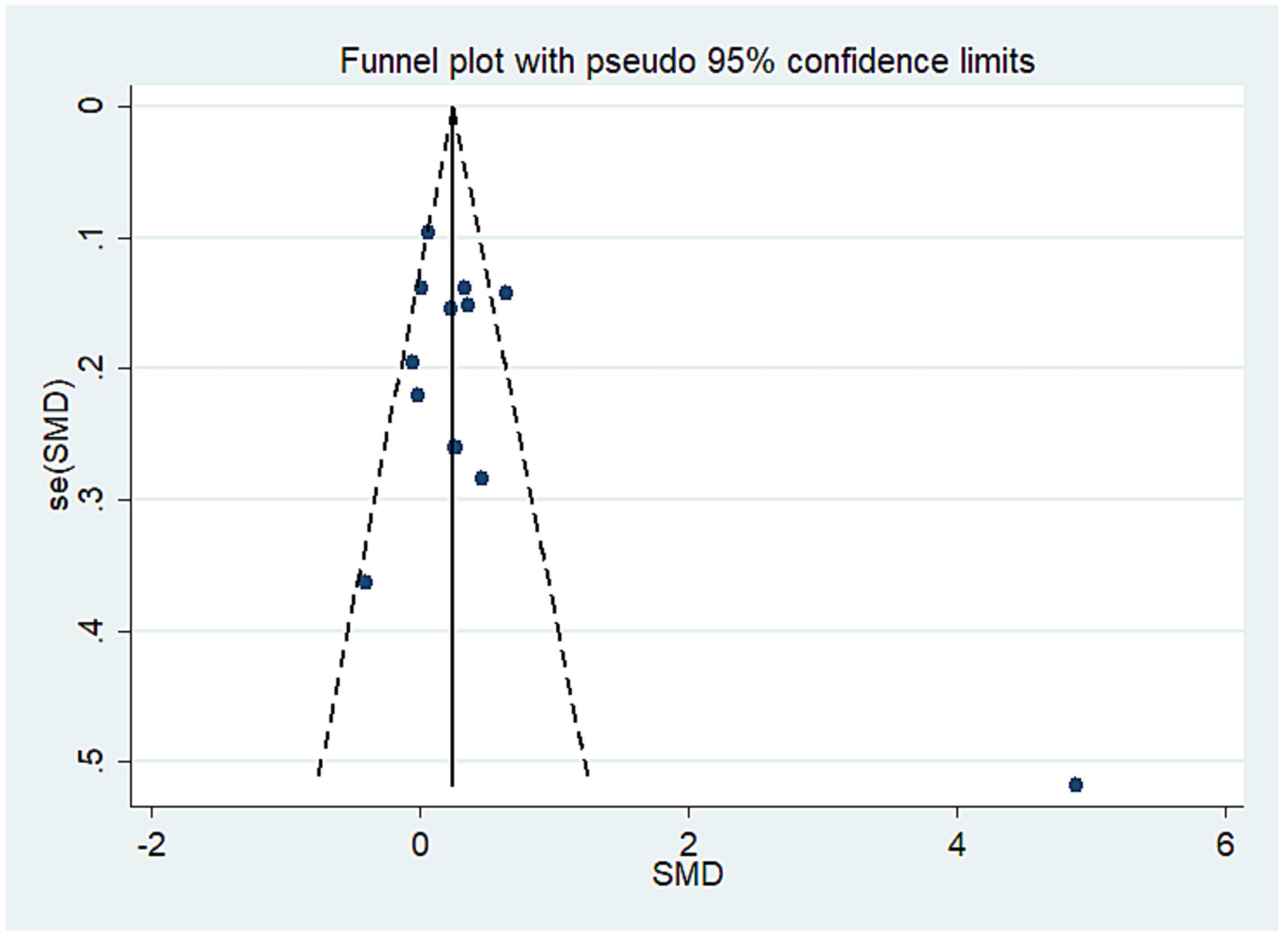
Literature review funnel plot based on primary outcome.

All 13 trials investigated the impact of exercise interventions delivered via telemedicine on pain in older adult patients with OA, comprising a telerehabilitation group (932 cases) and a control group (913 cases). The results indicated that exercise interventions provided through telemedicine were more effective than those in the control group, demonstrating a statistically significant SMD of 0.40 (SMD = 0.40, 95% CI: 0.11 to 0.69, *I*^2^ = 88.3% following heterogeneity adjustment, P_heterogeneity_ < 0.1; see Figure 2). Based on the GRADE evaluation, the level of quality of evidence was moderate (see Supplementary Figure 3). The funnel plot is symmetrically distributed (see Figure 3), suggesting no potential publication bias (P_egger_ = 0.14).

## Secondary outcome

### Effect of telemedicine-delivered exercise on physical function in older adult individuals with OA

**Figure 4:**
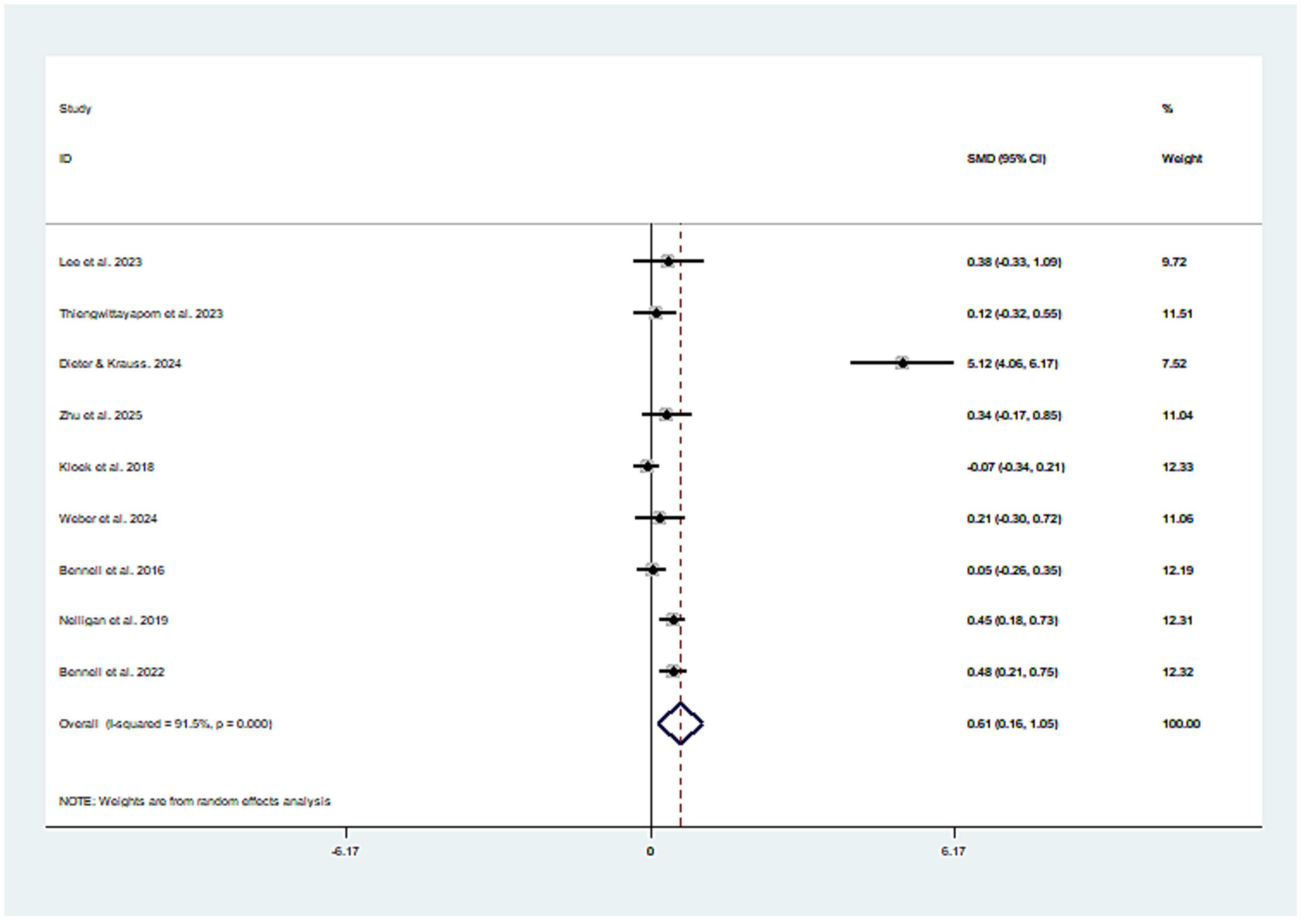
Literature review forest plot based on physical function.

**Figure 5:**
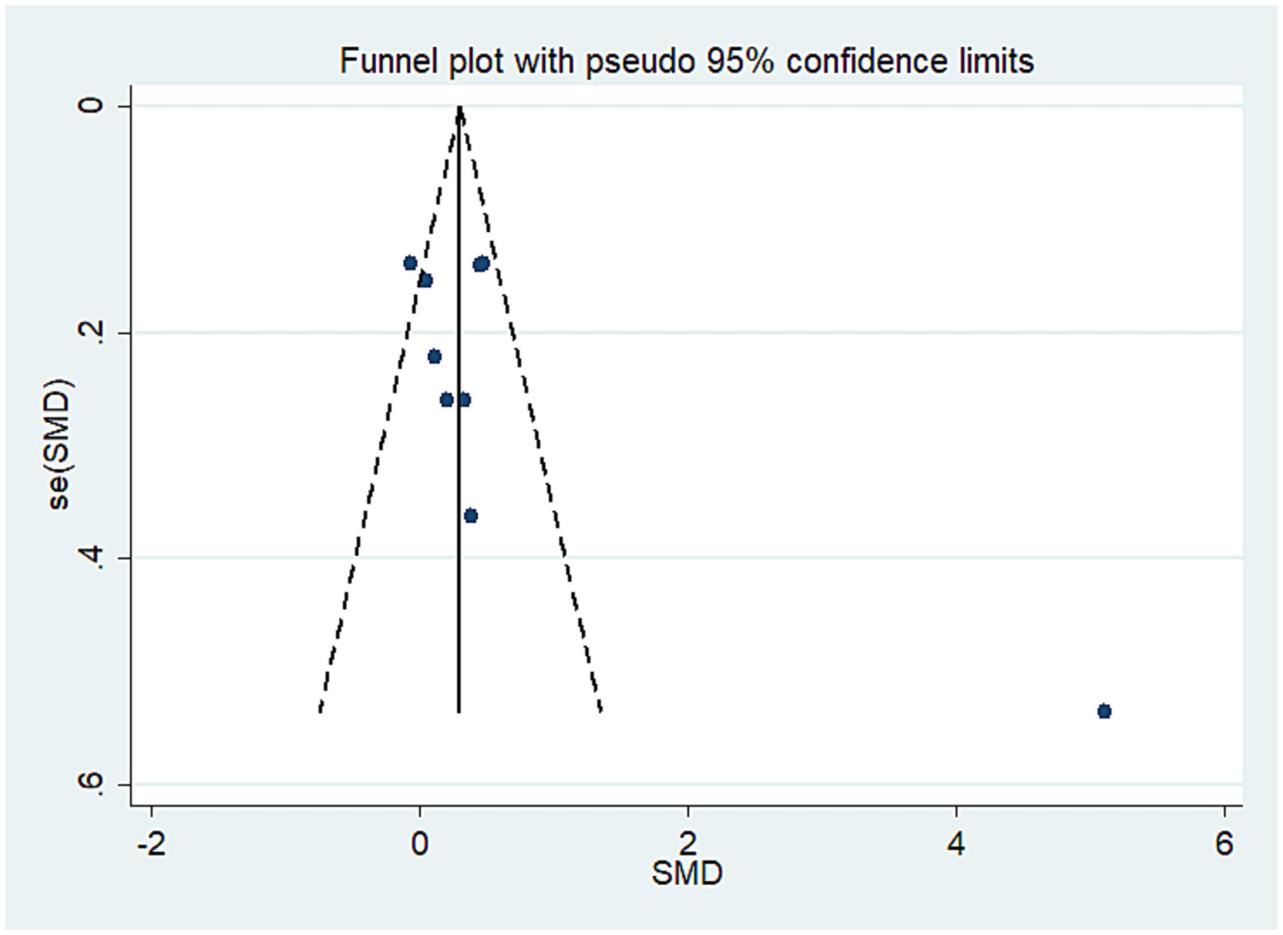
Literature review forest plot based on physical function.

Nine studies investigated the impact of mobile health technology-based exercise on physical function, involving a total of 1,088 participants. The results showed that participants in the mobile health exercise group had significantly improved physical function compared to the control group (SMD = 0.61, 95% CI: 0.16 to 1.05, *I*^2^ = 91.5%) (Figure 4). Additionally, the asymmetry in the funnel plot of dysfunction data indicated the absence of publication bias (P_egger_ = 0.09; see Figure 5).

### Effect of telemedicine-delivered exercise on quality of life in older adult individuals with OA

Data from 10 studies reported the impact of exercise delivered via telemedicine on quality of life, involving a total of 1,650 participants. The results indicated that the quality of life levels in the experimental group were significantly higher than those in the control group, with a SMD of 0.42 (95% CI: 0.07 to 0.77, *I*^2^ = 90.9%) (Supplementary Figure 4). Furthermore, the funnel plot revealed asymmetry in the quality of life data, suggesting the presence of publication bias (P_egger_ = 0.02) (Supplementary Figure 5).

### Subgroup analyses

Based on the primary outcome of pain, subgroup analyses were conducted considering various factors of interest. In the subgroup analysis focused on intervention duration, a total of 13 articles were included (see Table 2). Among these, 10 studies reported an intervention duration of 3 months or longer, while 3 studies had an intervention duration of less than 3 months. The analysis revealed that studies with an intervention duration of 3 months or more (SMD = 0.52, 95% CI: 0.18 to 0.87) demonstrated significantly greater pain improvement compared to those with an intervention duration of less than 3 months (SMD = 0.01, 95% CI: −0.31 to 0.33). Additionally, studies with a sample size of 100 or more (SMD = 0.23, 95% CI: 0.06 to 0.41) also exhibited significantly greater pain improvement compared to studies with a sample size of less than 100 (SMD = 0.84, 95% CI: −0.13 to 1.80). Furthermore, we conducted a subgroup analysis based on the form of intervention. The results indicated that exercise interventions, whether conducted via mobile phone (SMD = 0.32, 95% CI: 0.04 to 0.59), web (SMD = 0.34, 95% CI: 0.07 to 0.61), or app (SMD = 0.55, 95% CI: 0.02 to 1.07), were effective in reducing pain among older adult individuals with OA. Besides, Additionally, when conducting subgroup analyses on intervention duration and measurement methods, we found they explained the high heterogeneity in the study results. Studies with intervention durations exceeding three months showed higher heterogeneity (*I*^2^ = 90.80%), while those with durations under three months exhibited lower heterogeneity (*I*^2^ = 9%). Studies using HOOS or KOOS scales demonstrated higher heterogeneity (*I*^2^ = 93.80%), whereas those employing other assessment scales consistently showed lower heterogeneity.

**Table 2:** Primary results based on pain intensity and subgroup analyses.

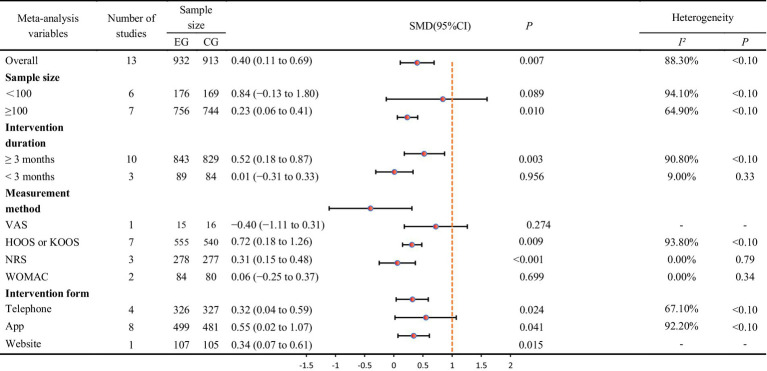

HOOS, Hip Disability and Osteoarthritis Outcome Score; KOOS, Knee Injury and Osteoarthritis Outcome Score; NRS, Numerical Rating Scale; VAS, Visual Analog Scale; WOMAC, Western Ontario and McMaster Universities Arthritis Index.

## Discussion

Based on 13 randomized controlled trials, this study confirms that older adult patients with osteoarthritis who participated in telemedicine exercise interventions exhibited significant improvements in pain levels, physical function, and quality of life when compared to both the conventional treatment group and the control group. Furthermore, the intervention effects were found to be more pronounced in studies where the sample size exceeded 100 participants and the duration of the intervention was longer than 3 months.

The remote exercise interventions for older adult individuals evaluated in this study can be categorized into three primary types: web-based remote exercise interventions, app-based remote exercise interventions, and smartphone-based remote exercise interventions. These interventions may comprise various combinations of exercise modalities, such as app-based remote exercise interventions supplemented by regular corrective actions and assessments conducted by rehabilitation therapists. Based on these three types of remote intervention forms, healthcare professionals must select the most suitable remote intervention type according to the patient’s actual condition in clinical practice.

The meta-analysis revealed that mobile health exercise interventions significantly reduced pain levels in older adult patients with OA, yielding a SMD of 0.40 (95% CI, 0.11 to 0.69). This finding aligns with prior research outcomes (39, 40). Dieter et al. (29) implemented a 12-week APP-based medical intervention via the re.flex mobile health system, delivering a progressive home exercise program to 61 patients with moderate-to-severe unicompartmental knee OA. Results showed the intervention group significantly outperformed the control in pain relief, knee function, and quality of life, with 92.5% adherence and no serious adverse events, confirming the intervention’s effectiveness and safety (29). A U. S.-based RCT included 103 adult knee OA patients meeting NICE criteria, implementing a 12-week telehealth intervention (physical therapy exercises, education, daily step goals) via Zoom, Fitbit, and ActiGraph GT3X. Versus the control group (only online resources), the intervention group showed significant improvements in knee pain relief, function, and quality of life (41). Several mechanisms may elucidate these outcomes. Firstly, the decline in physical functions among older adult individuals leads to reduced spatial mobility; however, the demand for exercise rehabilitation remains high, resulting in a significant gap between demand and availability (42). Remote exercise interventions, characterized by convenience and efficiency, significantly reduce the time costs of traveling to and from outpatient visits compared with traditional methods—rendering them highly operable and convenient, an advantage that is particularly prominent under extraordinary circumstances like the COVID-19 pandemic (43). Mobile health exercise interventions not only save time but also minimize crowding and reduce the risk of infection, all while maintaining the effectiveness of exercise interventions and ensuring individual health and safety (44). It is crucial to acknowledge that the proficiency of older adult individuals in utilizing telemedicine support devices is a vital factor, as not all seniors are adept at using smartphones or web applications. Particularly for low-income groups, research shows that currently 38% of older adult individuals still have relatively low smartphone usage rates (45). Therefore, when implementing remote exercise interventions, it is essential to consider the acceptability of support devices among older adult individuals, which also aids in enhancing compliance. Meanwhile, current studies have demonstrated that internet-based telemedicine can effectively enhance the self-efficacy of arthritis patients. This facilitates patients’ proactive adherence to exercise interventions, reduces dependence on healthcare providers, alleviates disease-related anxiety, and improves pain tolerance, thereby ensuring long-term intervention compliance (45).

Subgroup analysis revealed that studies with an intervention duration of at least three months demonstrated a more significant improvement in pain among older adult patients with OA. This is likely due to the pathological issues, such as muscle atrophy, which necessitate long-term interventions. Short-term interventions may only provide temporary symptom relief, whereas sustained interventions lasting over three months can gradually enhance peri-knee muscle strength, reduce inflammation, improve cartilage nutrition, and assist older adult patients in developing exercise habits, thereby increasing compliance (46). This shift facilitates a transition from mere symptomatic relief to actual pathological improvement. Furthermore, studies with a sample size of at least 100 participants yielded more significant results. This can be attributed to individual differences, such as age and comorbidities, which are prevalent among older adult patients. Larger sample sizes can mitigate random errors and enhance statistical power, in accordance with the law of large numbers, thus avoiding result deviations caused by extreme values in smaller samples and more accurately reflecting the true effect of the intervention on pain relief. Additionally, subgroup analysis based on the type of intervention indicated that exercise interventions delivered through applications, websites, or mobile phones effectively alleviated pain levels in older adult individuals with OA. This indicates that regardless of the form of remote exercise intervention, as long as the duration and quality of the intervention are adequately ensured, these interventions can effectively reduce pain levels in older adult (47).

### Strengths and limitations

This study represents the first meta-analysis focusing on the impact of mobile health exercise interventions on OA pain in older adult patients. The results demonstrate that mobile health exercise interventions significantly alleviate pain in older adult patients and effectively enhance their quality of life and physical function. Additionally, this intervention model offers substantial advantages for older adult individuals; leveraging its convenience and low cost, remote interventions can transcend geographical barriers, facilitating easier participation in intervention programs and thereby improving adherence and accessibility. Based on these research findings, this study provides important reference evidence for policymakers and clinicians regarding the form of exercise interventions, ultimately contributing to advancing subsequent related research and clinical practice applications, thereby benefiting more older adult patients with OA.

Several limitations of this study must be acknowledged. Firstly, some of the included studies that met the inclusion criteria exhibited methodological quality issues, such as a lack of blinding for participants or researchers, which were assessed to have a high risk of bias. This may compromise the internal validity and reliability of the study results. Meanwhile, this study exhibited substantial heterogeneity. Although subgroup analyses identified some sources of heterogeneity, the results should still be interpreted with caution. Secondly, the relatively limited number of studies included in the analysis somewhat undermines the robustness of the statistical analysis, potentially leading to insufficient representativeness of the overall effect. Furthermore, the studies included in this research involved diverse populations, with an average age of 60 years or older. This may have a significant impact on the results of our study. In future studies, the targeted selection of homogeneous populations for analysis may help reduce heterogeneity and enhance the precision of research findings. Besides, Including only published randomized controlled trials may lead to publication bias, thus our findings should be interpreted with caution. Additionally, although existing evidence has confirmed the positive effects of remote exercise interventions on pain relief in older adult patients with OA, clinical practice still requires further exploration and refinement in how to precisely formulate personalized exercise programs, scientifically design, and standardize the implementation of progressive exercise interventions to optimize therapeutic efficacy in future research.

## Conclusion

Findings from this study reveal that telemedicine-assisted exercise interventions significantly alleviate pain in older adult patients with OA. The analgesic effect is more pronounced when the intervention lasts beyond three months, under the condition that patient adherence and professional remote supervision are guaranteed. In terms of the practical value of the intervention, remote exercise interventions combine the advantages of high convenience and low cost, enabling older adult OA patients to more easily participate in the rehabilitation process, thereby achieving potentially favorable rehabilitation outcomes. It is important to note that future implementation of mobile health exercise interventions should focus on the acceptability of this intervention model among older adult individuals. Additionally, given the limitations inherent in this study and the yet-to-be-perfected nature of the mobile health exercise intervention model, the interpretation and application of the findings should be approached with caution.

## Data Availability

The original contributions presented in the study are included in the article/Supplementary material, further inquiries can be directed to the corresponding author.
