# Supplementary material for: Effectiveness of telemedicine-delivered exercise interventions in older adult patients with osteoarthritis: a systematic review and meta-analysis
Source: Front Public Health. 2025 Dec 3;13:1719841. doi: 10.3389/fpubh.2025.1719841 (PMC12708276; doi:10.3389/fpubh.2025.1719841)
Supplement: Supplementary file 1 [file Supplementary_file_1.docx]

**Cochrane library**

Search Name:

Date Run: 10/08/2025 16:59:03

Comment:

ID Search Hits

#1 Osteoarthritis OR Hand Osteoarthritis OR knee Osteoarthritis OR hip osteoarthritis OR knee arthrosis OR degenerative arthritis OR musculoskeletal pain OR chronic pain OR acute pain OR overuse pain OR chronic injury OR Nonspecific chronic pain OR Chronic widespread pain 105814

#2 Telemedicine OR mHealth OR Telehealth OR eHealth OR Tele-Intensive Care OR Telecare OR Tele-Care OR digital movement therapy OR digital movement therapies OR mobile health OR web-based intervention OR digital intervention OR computer-based intervention OR app-based intervention OR digital health application OR technology-assisted therapy OR technology-assisted therapies OR internet-based intervention OR computer-assisted therapy OR computer-assisted therapies OR health app OR mobile application OR smartphone OR online intervention OR internet-delivered intervention OR smartphone application OR sensors OR connected watch OR Telerehabilitation OR Internet OR Internet-Based Intervention OR Online OR App OR m-health OR mobile* OR apps OR application* OR internet rehabilitation OR web OR wearable OR mobile applications OR Internet-based OR web-based OR APP∗ 219635

#3 movement therapy OR movement therapies OR physical therapy OR physical therapies OR therapeutic exercise OR medical gymnastic OR traditional therapy OR traditional therapies OR manual therapy OR manual therapies OR physiotherapy OR training* OR sport OR strength* OR isometric* OR isotonic* OR isokinetic* OR aerobic* OR endurance OR weight* OR train* OR muscle strength* OR physical endurance OR therapy OR therapies OR rehabilitation OR physical activity OR physical exercise OR exercises OR activities, physical OR activity, physical OR physical activities OR exercise, physical OR exercises, physical OR physical exercises OR acute exercise OR acute exercises OR exercise, acute OR exercises, acute OR exercise, isometric OR exercises, isometric OR isometric exercises OR isometric exercise OR exercise, aerobic OR aerobic exercise OR aerobic exercises OR exercises, aerobic OR exercise training OR exercise trainings OR training, exercise OR trainings, exercise OR football OR tai chi OR basketball OR high-intensity interval training OR high intensity interval training OR high-intensity interval trainings OR interval training, high-intensity OR interval trainings, high-intensity OR training, high-intensity interval OR trainings, high-intensity interval OR high-intensity intermittent exercise OR exercise, high-intensity intermittent OR exercises, high-intensity intermittent OR high-intensity intermittent exercises OR sprint interval training OR sprint interval trainings 1240764

#4 Aged OR elderly OR older people OR older adults OR aged patient OR aged people OR aged person OR aged subject OR elderly patient OR elderly people OR elderly person OR elderly subject OR senior citizen 734889

#5 #1 and #2 and #3 and #4 7199

**Embase**

Session Results

.......................................................

No. Query Results Results Date

#1. ('osteoarthritis'/exp OR osteoarthritis OR 'hand 2,140 10 Aug 2025

osteoarthritis'/exp OR 'hand osteoarthritis' OR

(('hand'/exp OR hand) AND ('osteoarthritis'/exp

OR osteoarthritis)) OR 'knee osteoarthritis'/exp

OR 'knee osteoarthritis' OR (('knee'/exp OR knee)

AND ('osteoarthritis'/exp OR osteoarthritis)) OR

'hip osteoarthritis'/exp OR 'hip osteoarthritis'

OR (('hip'/exp OR hip) AND ('osteoarthritis'/exp

OR osteoarthritis)) OR 'knee arthrosis'/exp OR

'knee arthrosis' OR (('knee'/exp OR knee) AND

('arthrosis'/exp OR arthrosis)) OR 'degenerative

arthritis'/exp OR 'degenerative arthritis' OR

(degenerative AND ('arthritis'/exp OR arthritis))

OR 'musculoskeletal pain'/exp OR 'musculoskeletal

pain' OR (musculoskeletal AND ('pain'/exp OR

pain)) OR 'chronic pain'/exp OR 'chronic pain' OR

(chronic AND ('pain'/exp OR pain)) OR 'acute

pain'/exp OR 'acute pain' OR (acute AND

('pain'/exp OR pain)) OR 'overuse pain' OR

(overuse AND ('pain'/exp OR pain)) OR 'chronic

injury' OR (chronic AND ('injury'/exp OR injury))

OR 'nonspecific chronic pain' OR (nonspecific AND

chronic AND ('pain'/exp OR pain)) OR 'chronic

widespread pain'/exp OR 'chronic widespread pain'

OR (chronic AND widespread AND ('pain'/exp OR

pain))) AND (telemedicine:ab,ti OR mhealth:ab,ti

OR telehealth:ab,ti OR ehealth:ab,ti OR

'tele-intensive care':ab,ti OR telecare:ab,ti OR

'tele care':ab,ti OR 'digital movement

therapy':ab,ti OR 'digital movement

therapies':ab,ti OR 'mobile health':ab,ti OR

'web-based intervention':ab,ti OR 'digital

intervention':ab,ti OR 'computer-based

intervention':ab,ti OR 'app-based

intervention':ab,ti OR 'digital health

application':ab,ti OR 'technology-assisted

therapy':ab,ti OR 'technology-assisted

therapies':ab,ti OR 'computer-assisted

therapy':ab,ti OR 'computer-assisted

therapies':ab,ti OR 'health app':ab,ti OR 'mobile

application':ab,ti OR smartphone:ab,ti OR 'online

intervention':ab,ti OR 'internet-delivered

intervention':ab,ti OR 'smartphone

application':ab,ti OR sensors:ab,ti OR 'connected

watch':ab,ti OR telerehabilitation:ab,ti OR

internet:ab,ti OR 'internet-based

intervention':ab,ti OR online:ab,ti OR app:ab,ti

OR 'm health':ab,ti OR mobile*:ab,ti OR

apps:ab,ti OR application*:ab,ti OR 'internet

rehabilitation':ab,ti OR web:ab,ti OR

wearable:ab,ti OR 'mobile applications':ab,ti OR

'internet based':ab,ti OR 'web based':ab,ti OR

app∗:ab,ti) AND ('movement therapy':ab,ti OR

'movement therapies':ab,ti OR 'physical

therapy':ab,ti OR 'physical therapies':ab,ti OR

'therapeutic exercise':ab,ti OR 'medical

gymnastic':ab,ti OR 'traditional therapy':ab,ti

OR 'traditional therapies':ab,ti OR 'manual

therapy':ab,ti OR 'manual therapies':ab,ti OR

physiotherapy:ab,ti OR training*:ab,ti OR

sport:ab,ti OR strength*:ab,ti OR

isometric*:ab,ti OR isotonic*:ab,ti OR

isokinetic*:ab,ti OR aerobic*:ab,ti OR

endurance:ab,ti OR weight*:ab,ti OR train*:ab,ti

OR 'muscle strength*':ab,ti OR 'physical

endurance':ab,ti OR therapy:ab,ti OR

therapies:ab,ti OR rehabilitation:ab,ti OR

'physical activity':ab,ti OR 'physical

exercise':ab,ti OR exercises:ab,ti OR

'activities, physical':ab,ti OR 'activity,

physical':ab,ti OR 'physical activities':ab,ti OR

'exercise, physical':ab,ti OR 'exercises,

physical':ab,ti OR 'physical exercises':ab,ti OR

'acute exercise':ab,ti OR 'acute exercises':ab,ti

OR 'exercise, acute':ab,ti OR 'exercises,

acute':ab,ti OR 'exercise, isometric':ab,ti OR

'exercises, isometric':ab,ti OR 'isometric

exercises':ab,ti OR 'isometric exercise':ab,ti OR

'exercise, aerobic':ab,ti OR 'aerobic

exercise':ab,ti OR 'aerobic exercises':ab,ti OR

'exercises, aerobic':ab,ti OR 'exercise

training':ab,ti OR 'exercise trainings':ab,ti OR

'training, exercise':ab,ti OR 'trainings,

exercise':ab,ti OR football:ab,ti OR 'tai

chi':ab,ti OR basketball:ab,ti OR 'high-intensity

interval training':ab,ti OR 'high intensity

interval training':ab,ti OR 'high-intensity

interval trainings':ab,ti OR 'interval training,

high-intensity':ab,ti OR 'interval trainings,

high-intensity':ab,ti OR 'training,

high-intensity interval':ab,ti OR 'trainings,

high-intensity interval':ab,ti OR 'high-intensity

intermittent exercise':ab,ti OR 'exercise,

high-intensity intermittent':ab,ti OR 'exercises,

high-intensity intermittent':ab,ti OR

'high-intensity intermittent exercises':ab,ti OR

'sprint interval training':ab,ti OR 'sprint

interval trainings':ab,ti) AND (aged:ab,ti OR

elderly:ab,ti OR 'older people':ab,ti OR 'older

adults':ab,ti OR 'aged patient':ab,ti OR 'aged

people':ab,ti OR 'aged person':ab,ti OR 'aged

subject':ab,ti OR 'elderly patient':ab,ti OR

'elderly people':ab,ti OR 'elderly person':ab,ti

OR 'elderly subject':ab,ti OR 'senior

citizen':ab,ti) AND ('double blind*':ab,ti OR

'single blind*':ab,ti OR placebo*:ab,ti OR

random*:ab,ti OR 'prospective stud*':ab,ti OR

'follow-up stud*':ab,ti OR 'controlled

trial*':ab,ti OR 'evaluation stud*':ab,ti OR

'comparative stud*':ab,ti OR 'research

design':ab,ti OR 'clinical trial*':ab,ti)

.......................................................

**Pubmed**

| Search number | Query | Sort By | Filters | Search Details | Results | Time | Date |
| --- | --- | --- | --- | --- | --- | --- | --- |
| 6 | ((((movement therapy[Title/Abstract] OR movement therapies[Title/Abstract] OR physical therapy[Title/Abstract] OR physical therapies[Title/Abstract] OR therapeutic exercise[Title/Abstract] OR medical gymnastic[Title/Abstract] OR traditional therapy[Title/Abstract] OR traditional therapies[Title/Abstract] OR manual therapy[Title/Abstract] OR manual therapies[Title/Abstract] OR physiotherapy[Title/Abstract] OR exercis*[Title/Abstract] OR exertion*[Title/Abstract] OR training*[Title/Abstract] OR sport[Title/Abstract] OR strength*[Title/Abstract] OR isometric*[Title/Abstract] OR isotonic*[Title/Abstract] OR isokinetic*[Title/Abstract] OR aerobic*[Title/Abstract] OR endurance[Title/Abstract] OR weight*[Title/Abstract] OR exercis*[Title/Abstract] OR train*[Title/Abstract] OR muscle strength*[Title/Abstract] OR physical endurance[Title/Abstract] OR physiotherap*[Title/Abstract] OR therapy[Title/Abstract] OR therapies[Title/Abstract] OR rehabilitation[Title/Abstract] OR physical activity[Title/Abstract] OR physical exercise[Title/Abstract] OR exercises[Title/Abstract] OR physical activity[Title/Abstract] OR activities, physical[Title/Abstract] OR activity, physical[Title/Abstract] OR physical activities[Title/Abstract] OR exercise, physical[Title/Abstract] OR exercises, physical[Title/Abstract] OR physical exercise[Title/Abstract] OR physical exercises[Title/Abstract] OR acute exercise[Title/Abstract] OR acute exercises[Title/Abstract] OR exercise, acute[Title/Abstract] OR exercises, acute[Title/Abstract] OR exercise, isometric[Title/Abstract] OR exercises, isometric[Title/Abstract] OR isometric exercises[Title/Abstract] OR isometric exercise[Title/Abstract] OR exercise, aerobic[Title/Abstract] OR aerobic exercise[Title/Abstract] OR aerobic exercises[Title/Abstract] OR exercises, aerobic[Title/Abstract] OR exercise training[Title/Abstract] OR exercise trainings[Title/Abstract] OR training, exercise[Title/Abstract] OR trainings, exercise[Title/Abstract] OR football:ti,ab,kw[Title/Abstract] OR tai chi[Title/Abstract] OR basketball:ti,ab,kw[Title/Abstract] OR high-intensity interval training[Title/Abstract] OR high intensity interval training[Title/Abstract] OR high-intensity interval trainings[Title/Abstract] OR interval training, high-intensity[Title/Abstract] OR interval trainings, high-intensity[Title/Abstract] OR training, high-intensity interval[Title/Abstract] OR trainings, high-intensity interval[Title/Abstract] OR high-intensity intermittent exercise[Title/Abstract] OR exercise, high-intensity intermittent[Title/Abstract] OR exercises, high-intensity intermittent[Title/Abstract] OR high-intensity intermittent exercises[Title/Abstract] OR sprint interval training[Title/Abstract] OR sprint interval trainings[Title/Abstract]) AND (Virtual Medicine Medicine[Title/Abstract] OR Virtual Tele-Referral[Title/Abstract] OR Tele Referral[Title/Abstract] OR Tele-Referrals[Title/Abstract] OR Mobile Health[Title/Abstract] OR mHealth[Title/Abstract] OR Telehealth[Title/Abstract] OR eHealth[Title/Abstract] OR Tele-Intensive Care[Title/Abstract] OR Tele Intensive Care[Title/Abstract] OR Tele-ICU[Title/Abstract] OR Tele ICU[Title/Abstract] OR Telecare[Title/Abstract] OR Tele-Care[Title/Abstract] OR Tele Care[Title/Abstract] OR digital movement therapy[Title/Abstract] OR digital movement therapies[Title/Abstract] OR mobile health[Title/Abstract] OR etherapy[Title/Abstract] OR etherapies[Title/Abstract] OR web-based intervention[Title/Abstract] OR digital intervention[Title/Abstract] OR computer-based intervention[Title/Abstract] OR app-based intervention[Title/Abstract] OR digital health application[Title/Abstract] OR technology-assisted therapy[Title/Abstract] OR technology-assisted therapies[Title/Abstract] OR internet-based intervention[Title/Abstract] OR computer-assisted therapy[Title/Abstract] OR computer-assisted therapies[Title/Abstract] OR health app[Title/Abstract] OR mobile application[Title/Abstract] OR smartphone[Title/Abstract] OR telemedicine[Title/Abstract] OR online intervention[Title/Abstract] OR internet-delivered intervention[Title/Abstract] OR mobile application[Title/Abstract] OR smartphone application[Title/Abstract] OR sensors[Title/Abstract] OR connected watch[Title/Abstract] OR Telemedicine[Title/Abstract] OR Telerehabilitation[Title/Abstract] OR Internet[Title/Abstract] OR Internet-Based Intervention[Title/Abstract] OR Online[Title/Abstract] OR App[Title/Abstract] OR m-health[Title/Abstract] OR smartphone[Title/Abstract] OR mobile*[Title/Abstract] OR iphone[Title/Abstract] OR ipad[Title/Abstract] OR app[Title/Abstract] OR apps[Title/Abstract] OR application*[Title/Abstract] OR telemedicine[Title/Abstract] OR telerehabilitation[Title/Abstract] OR internet rehabilitation[Title/Abstract] OR web[Title/Abstract] OR online[Title/Abstract] OR app[Title/Abstract] OR wearable[Title/Abstract] OR sensor[Title/Abstract] OR mobile applications[Title/Abstract] OR telemedicine[Title/Abstract] OR Internet-based[Title/Abstract] OR web-based[Title/Abstract] OR APP∗[Title/Abstract])) AND (((((((((((double blind*[Title/Abstract]) OR (single blind*[Title/Abstract])) OR (placebo*[Title/Abstract])) OR (random*[Title/Abstract])) OR (prospective stud*[Title/Abstract])) OR (follow-up stud*[Title/Abstract])) OR (controlled trial*[Title/Abstract])) OR (evaluation stud*[Title/Abstract])) OR (comparative stud*[Title/Abstract])) OR (research design[Title/Abstract])) OR (clinical trial*[Title/Abstract]))) AND (Osteoarthritis[Title/Abstract] OR Hip Osteoarthritis [Title/Abstract] OR Hand Osteoarthritis[Title/Abstract] OR knee Osteoarthritis[Title/Abstract] OR knee arthrosis[Title/Abstract] OR knee cartilage[Title/Abstract] OR degenerative arthritis[Title/Abstract] OR musculoskeletal pain[Title/Abstract] OR chronic pain[Title/Abstract] OR overuse pain[Title/Abstract] OR chronic injury[Title/Abstract] OR chronic injuries knee[Title/Abstract] OR Nonspecific chronic pain[Title/Abstract] OR Chronic widespread pain[Title/Abstract] "Osteoarthritis"[Title/Abstract] OR "knee osteoarthritis"[Title/Abstract] OR "knee arthrosis"[Title/Abstract] OR "knee cartilage"[Title/Abstract] OR "degenerative arthritis"[Title/Abstract] OR "musculoskeletal pain"[Title/Abstract] OR "chronic pain"[Title/Abstract] OR "acute pain"[Title/Abstract] OR "overuse pain"[Title/Abstract] OR "chronic injury"[Title/Abstract] OR (("Chronic"[All Fields] OR "chronical"[All Fields] OR "chronically"[All Fields] OR "chronicities"[All Fields] OR "chronicity"[All Fields] OR "chronicization"[All Fields] OR "chronics"[All Fields]) AND "injuries knee"[Title/Abstract]) OR "nonspecific chronic pain"[Title/Abstract] OR "chronic widespread pain"[Title/Abstract])) AND ((((((((((((((Aged[Title/Abstract]) OR (elderly[Title/Abstract])) OR (older people[Title/Abstract])) OR (older adults[Title/Abstract])) OR (aged patient[Title/Abstract])) OR (aged people[Title/Abstract])) OR (aged person[Title/Abstract])) OR (aged subject[Title/Abstract])) OR (elderly patient[Title/Abstract])) OR (elderly people[Title/Abstract])) OR (elderly person[Title/Abstract])) OR (elderly subject[Title/Abstract])) OR (senior citizen[Title/Abstract])) OR (senium[Title/Abstract]) "Aged"[Title/Abstract] OR "elderly"[Title/Abstract] OR "older people"[Title/Abstract] OR "older adults"[Title/Abstract] OR "aged patient"[Title/Abstract] OR "aged people"[Title/Abstract] OR "aged person"[Title/Abstract] OR "aged subject"[Title/Abstract] OR "elderly patient"[Title/Abstract] OR "elderly people"[Title/Abstract] OR "elderly person"[Title/Abstract] OR "elderly subject"[Title/Abstract] OR "senior citizen"[Title/Abstract] OR "senium"[Title/Abstract]) |  |  | ("movement therapy"[Title/Abstract] OR "movement therapies"[Title/Abstract] OR "physical therapy"[Title/Abstract] OR "physical therapies"[Title/Abstract] OR "therapeutic exercise"[Title/Abstract] OR "medical gymnastic"[Title/Abstract] OR "traditional therapy"[Title/Abstract] OR "traditional therapies"[Title/Abstract] OR "manual therapy"[Title/Abstract] OR "manual therapies"[Title/Abstract] OR "physiotherapy"[Title/Abstract] OR "exercis*"[Title/Abstract] OR "exertion*"[Title/Abstract] OR "training*"[Title/Abstract] OR "sport"[Title/Abstract] OR "strength*"[Title/Abstract] OR "isometric*"[Title/Abstract] OR "isotonic*"[Title/Abstract] OR "isokinetic*"[Title/Abstract] OR "aerobic*"[Title/Abstract] OR "endurance"[Title/Abstract] OR "weight*"[Title/Abstract] OR "exercis*"[Title/Abstract] OR "train*"[Title/Abstract] OR "muscle strength*"[Title/Abstract] OR "physical endurance"[Title/Abstract] OR "physiotherap*"[Title/Abstract] OR "therapy"[Title/Abstract] OR "therapies"[Title/Abstract] OR "rehabilitation"[Title/Abstract] OR "physical activity"[Title/Abstract] OR "physical exercise"[Title/Abstract] OR "exercises"[Title/Abstract] OR "physical activity"[Title/Abstract] OR "activities physical"[Title/Abstract] OR "activity physical"[Title/Abstract] OR "physical activities"[Title/Abstract] OR "exercise physical"[Title/Abstract] OR "exercises physical"[Title/Abstract] OR "physical exercise"[Title/Abstract] OR "physical exercises"[Title/Abstract] OR "acute exercise"[Title/Abstract] OR "acute exercises"[Title/Abstract] OR "exercise acute"[Title/Abstract] OR "exercises acute"[Title/Abstract] OR "exercise isometric"[Title/Abstract] OR "exercises isometric"[Title/Abstract] OR "isometric exercises"[Title/Abstract] OR "isometric exercise"[Title/Abstract] OR "exercise aerobic"[Title/Abstract] OR "aerobic exercise"[Title/Abstract] OR "aerobic exercises"[Title/Abstract] OR "exercises aerobic"[Title/Abstract] OR "exercise training"[Title/Abstract] OR "exercise trainings"[Title/Abstract] OR "training exercise"[Title/Abstract] OR (("education"[MeSH Subheading] OR "education"[All Fields] OR "training"[All Fields] OR "education"[MeSH Terms] OR "train"[All Fields] OR "train s"[All Fields] OR "trained"[All Fields] OR "training s"[All Fields] OR "trainings"[All Fields] OR "trains"[All Fields]) AND "exercise"[Title/Abstract]) OR ((("football"[MeSH Terms] OR "football"[All Fields] OR "football s"[All Fields] OR "footballer"[All Fields] OR "footballer s"[All Fields] OR "footballers"[All Fields] OR "footballs"[All Fields]) AND "ti ab"[All Fields]) AND "kw"[Title/Abstract]) OR "tai chi"[Title/Abstract] OR ((("basketball"[MeSH Terms] OR "basketball"[All Fields] OR "basketballs"[All Fields] OR "basketballers"[All Fields]) AND "ti ab"[All Fields]) AND "kw"[Title/Abstract]) OR "high intensity interval training"[Title/Abstract] OR "high intensity interval training"[Title/Abstract] OR "high intensity interval trainings"[Title/Abstract] OR "interval training high intensity"[Title/Abstract] OR ((("interval"[All Fields] OR "intervals"[All Fields]) AND ("education"[MeSH Subheading] OR "education"[All Fields] OR "training"[All Fields] OR "education"[MeSH Terms] OR "train"[All Fields] OR "train s"[All Fields] OR "trained"[All Fields] OR "training s"[All Fields] OR "trainings"[All Fields] OR "trains"[All Fields])) AND "high-intensity"[Title/Abstract]) OR "training high intensity interval"[Title/Abstract] OR (("education"[MeSH Subheading] OR "education"[All Fields] OR "training"[All Fields] OR "education"[MeSH Terms] OR "train"[All Fields] OR "train s"[All Fields] OR "trained"[All Fields] OR "training s"[All Fields] OR "trainings"[All Fields] OR "trains"[All Fields]) AND "high intensity interval"[Title/Abstract]) OR "high intensity intermittent exercise"[Title/Abstract] OR "exercise high intensity intermittent"[Title/Abstract] OR (("exercise"[MeSH Terms] OR "exercise"[All Fields] OR "exercises"[All Fields] OR "exercise therapy"[MeSH Terms] OR ("exercise"[All Fields] AND "therapy"[All Fields]) OR "exercise therapy"[All Fields] OR "exercising"[All Fields] OR "exercise s"[All Fields] OR "exercised"[All Fields] OR "exerciser"[All Fields] OR "exercisers"[All Fields]) AND "high intensity intermittent"[Title/Abstract]) OR "high intensity intermittent exercises"[Title/Abstract] OR "sprint interval training"[Title/Abstract] OR "sprint interval trainings"[Title/Abstract]) AND ((("virtual"[All Fields] OR "virtuality"[All Fields] OR "virtualization"[All Fields] OR "virtualized"[All Fields] OR "virtualizing"[All Fields] OR "virtuals"[All Fields]) AND "medicine medicine"[Title/Abstract]) OR (("virtual"[All Fields] OR "virtuality"[All Fields] OR "virtualization"[All Fields] OR "virtualized"[All Fields] OR "virtualizing"[All Fields] OR "virtuals"[All Fields]) AND "Tele-Referral"[Title/Abstract]) OR "Tele-Referral"[Title/Abstract] OR "Tele-Referrals"[Title/Abstract] OR "mobile health"[Title/Abstract] OR "mHealth"[Title/Abstract] OR "Telehealth"[Title/Abstract] OR "eHealth"[Title/Abstract] OR "tele intensive care"[Title/Abstract] OR "tele intensive care"[Title/Abstract] OR "Tele-ICU"[Title/Abstract] OR "Tele-ICU"[Title/Abstract] OR "Telecare"[Title/Abstract] OR "Tele-Care"[Title/Abstract] OR "Tele-Care"[Title/Abstract] OR (("digitalisation"[All Fields] OR "digitalised"[All Fields] OR "digitalization"[All Fields] OR "digitalize"[All Fields] OR "digitalized"[All Fields] OR "digitalizer"[All Fields] OR "digitalizing"[All Fields] OR "digitally"[All Fields] OR "digitals"[All Fields] OR "digitization"[All Fields] OR "digitizations"[All Fields] OR "digitize"[All Fields] OR "digitized"[All Fields] OR "digitizer"[All Fields] OR "digitizers"[All Fields] OR "digitizes"[All Fields] OR "digitizing"[All Fields] OR "radiographic image enhancement"[MeSH Terms] OR ("radiographic"[All Fields] AND "image"[All Fields] AND "enhancement"[All Fields]) OR "radiographic image enhancement"[All Fields] OR "digital"[All Fields]) AND "movement therapy"[Title/Abstract]) OR (("digitalisation"[All Fields] OR "digitalised"[All Fields] OR "digitalization"[All Fields] OR "digitalize"[All Fields] OR "digitalized"[All Fields] OR "digitalizer"[All Fields] OR "digitalizing"[All Fields] OR "digitally"[All Fields] OR "digitals"[All Fields] OR "digitization"[All Fields] OR "digitizations"[All Fields] OR "digitize"[All Fields] OR "digitized"[All Fields] OR "digitizer"[All Fields] OR "digitizers"[All Fields] OR "digitizes"[All Fields] OR "digitizing"[All Fields] OR "radiographic image enhancement"[MeSH Terms] OR ("radiographic"[All Fields] AND "image"[All Fields] AND "enhancement"[All Fields]) OR "radiographic image enhancement"[All Fields] OR "digital"[All Fields]) AND "movement therapies"[Title/Abstract]) OR "mobile health"[Title/Abstract] OR "etherapy"[Title/Abstract] OR "etherapies"[Title/Abstract] OR "web based intervention"[Title/Abstract] OR "digital intervention"[Title/Abstract] OR "computer based intervention"[Title/Abstract] OR "app based intervention"[Title/Abstract] OR "digital health application"[Title/Abstract] OR "technology assisted therapy"[Title/Abstract] OR "technology assisted therapies"[Title/Abstract] OR "internet based intervention"[Title/Abstract] OR "computer assisted therapy"[Title/Abstract] OR "computer assisted therapies"[Title/Abstract] OR "health app"[Title/Abstract] OR "mobile application"[Title/Abstract] OR "smartphone"[Title/Abstract] OR "Telemedicine"[Title/Abstract] OR "online intervention"[Title/Abstract] OR "internet delivered intervention"[Title/Abstract] OR "mobile application"[Title/Abstract] OR "smartphone application"[Title/Abstract] OR "sensors"[Title/Abstract] OR "connected watch"[Title/Abstract] OR "Telemedicine"[Title/Abstract] OR "Telerehabilitation"[Title/Abstract] OR "Internet"[Title/Abstract] OR "internet based intervention"[Title/Abstract] OR "Online"[Title/Abstract] OR "app"[Title/Abstract] OR "m-health"[Title/Abstract] OR "smartphone"[Title/Abstract] OR "mobile*"[Title/Abstract] OR "iphone"[Title/Abstract] OR "ipad"[Title/Abstract] OR "app"[Title/Abstract] OR "apps"[Title/Abstract] OR "application*"[Title/Abstract] OR "Telemedicine"[Title/Abstract] OR "Telerehabilitation"[Title/Abstract] OR "internet rehabilitation"[Title/Abstract] OR "web"[Title/Abstract] OR "Online"[Title/Abstract] OR "app"[Title/Abstract] OR "wearable"[Title/Abstract] OR "sensor"[Title/Abstract] OR "mobile applications"[Title/Abstract] OR "Telemedicine"[Title/Abstract] OR "internet based"[Title/Abstract] OR "web-based"[Title/Abstract] OR "app"[Title/Abstract]) AND ("double blind*"[Title/Abstract] OR "single blind*"[Title/Abstract] OR "placebo*"[Title/Abstract] OR "random*"[Title/Abstract] OR "prospective stud*"[Title/Abstract] OR "follow up stud*"[Title/Abstract] OR "controlled trial*"[Title/Abstract] OR "evaluation stud*"[Title/Abstract] OR "comparative stud*"[Title/Abstract] OR "research design"[Title/Abstract] OR "clinical trial*"[Title/Abstract]) AND ((("Osteoarthritis"[Title/Abstract] OR "hip osteoarthritis"[Title/Abstract] OR "hand osteoarthritis"[Title/Abstract] OR "knee osteoarthritis"[Title/Abstract] OR "knee arthrosis"[Title/Abstract] OR "knee cartilage"[Title/Abstract] OR "degenerative arthritis"[Title/Abstract] OR "musculoskeletal pain"[Title/Abstract] OR "chronic pain"[Title/Abstract] OR "overuse pain"[Title/Abstract] OR "chronic injury"[Title/Abstract] OR (("Chronic"[All Fields] OR "chronical"[All Fields] OR "chronically"[All Fields] OR "chronicities"[All Fields] OR "chronicity"[All Fields] OR "chronicization"[All Fields] OR "chronics"[All Fields]) AND "injuries knee"[Title/Abstract]) OR "nonspecific chronic pain"[Title/Abstract] OR "chronic widespread pain"[Title/Abstract]) AND "Osteoarthritis"[Title/Abstract]) OR "knee osteoarthritis"[Title/Abstract] OR "knee arthrosis"[Title/Abstract] OR "knee cartilage"[Title/Abstract] OR "degenerative arthritis"[Title/Abstract] OR "musculoskeletal pain"[Title/Abstract] OR "chronic pain"[Title/Abstract] OR "acute pain"[Title/Abstract] OR "overuse pain"[Title/Abstract] OR "chronic injury"[Title/Abstract] OR (("Chronic"[All Fields] OR "chronical"[All Fields] OR "chronically"[All Fields] OR "chronicities"[All Fields] OR "chronicity"[All Fields] OR "chronicization"[All Fields] OR "chronics"[All Fields]) AND "injuries knee"[Title/Abstract]) OR "nonspecific chronic pain"[Title/Abstract] OR "chronic widespread pain"[Title/Abstract]) AND ((("Aged"[Title/Abstract] OR "elderly"[Title/Abstract] OR "older people"[Title/Abstract] OR "older adults"[Title/Abstract] OR "aged patient"[Title/Abstract] OR "aged people"[Title/Abstract] OR "aged person"[Title/Abstract] OR "aged subject"[Title/Abstract] OR "elderly patient"[Title/Abstract] OR "elderly people"[Title/Abstract] OR "elderly person"[Title/Abstract] OR "elderly subject"[Title/Abstract] OR "senior citizen"[Title/Abstract] OR "senium"[Title/Abstract]) AND "Aged"[Title/Abstract]) OR "elderly"[Title/Abstract] OR "older people"[Title/Abstract] OR "older adults"[Title/Abstract] OR "aged patient"[Title/Abstract] OR "aged people"[Title/Abstract] OR "aged person"[Title/Abstract] OR "aged subject"[Title/Abstract] OR "elderly patient"[Title/Abstract] OR "elderly people"[Title/Abstract] OR "elderly person"[Title/Abstract] OR "elderly subject"[Title/Abstract] OR "senior citizen"[Title/Abstract] OR "senium"[Title/Abstract]) | 274 | 6:04:56 | 2025/8/10 |
| 5 | (((((((((((((Aged[Title/Abstract]) OR (elderly[Title/Abstract])) OR (older people[Title/Abstract])) OR (older adults[Title/Abstract])) OR (aged patient[Title/Abstract])) OR (aged people[Title/Abstract])) OR (aged person[Title/Abstract])) OR (aged subject[Title/Abstract])) OR (elderly patient[Title/Abstract])) OR (elderly people[Title/Abstract])) OR (elderly person[Title/Abstract])) OR (elderly subject[Title/Abstract])) OR (senior citizen[Title/Abstract])) OR (senium[Title/Abstract]) "Aged"[Title/Abstract] OR "elderly"[Title/Abstract] OR "older people"[Title/Abstract] OR "older adults"[Title/Abstract] OR "aged patient"[Title/Abstract] OR "aged people"[Title/Abstract] OR "aged person"[Title/Abstract] OR "aged subject"[Title/Abstract] OR "elderly patient"[Title/Abstract] OR "elderly people"[Title/Abstract] OR "elderly person"[Title/Abstract] OR "elderly subject"[Title/Abstract] OR "senior citizen"[Title/Abstract] OR "senium"[Title/Abstract] |  |  | (("Aged"[Title/Abstract] OR "elderly"[Title/Abstract] OR "older people"[Title/Abstract] OR "older adults"[Title/Abstract] OR "aged patient"[Title/Abstract] OR "aged people"[Title/Abstract] OR "aged person"[Title/Abstract] OR "aged subject"[Title/Abstract] OR "elderly patient"[Title/Abstract] OR "elderly people"[Title/Abstract] OR "elderly person"[Title/Abstract] OR "elderly subject"[Title/Abstract] OR "senior citizen"[Title/Abstract] OR "senium"[Title/Abstract]) AND "Aged"[Title/Abstract]) OR "elderly"[Title/Abstract] OR "older people"[Title/Abstract] OR "older adults"[Title/Abstract] OR "aged patient"[Title/Abstract] OR "aged people"[Title/Abstract] OR "aged person"[Title/Abstract] OR "aged subject"[Title/Abstract] OR "elderly patient"[Title/Abstract] OR "elderly people"[Title/Abstract] OR "elderly person"[Title/Abstract] OR "elderly subject"[Title/Abstract] OR "senior citizen"[Title/Abstract] OR "senium"[Title/Abstract] | 1,232,201 | 6:04:26 | 2025/8/10 |
| 4 | Osteoarthritis[Title/Abstract] OR Hip Osteoarthritis [Title/Abstract] OR Hand Osteoarthritis[Title/Abstract] OR knee Osteoarthritis[Title/Abstract] OR knee arthrosis[Title/Abstract] OR knee cartilage[Title/Abstract] OR degenerative arthritis[Title/Abstract] OR musculoskeletal pain[Title/Abstract] OR chronic pain[Title/Abstract] OR overuse pain[Title/Abstract] OR chronic injury[Title/Abstract] OR chronic injuries knee[Title/Abstract] OR Nonspecific chronic pain[Title/Abstract] OR Chronic widespread pain[Title/Abstract] "Osteoarthritis"[Title/Abstract] OR "knee osteoarthritis"[Title/Abstract] OR "knee arthrosis"[Title/Abstract] OR "knee cartilage"[Title/Abstract] OR "degenerative arthritis"[Title/Abstract] OR "musculoskeletal pain"[Title/Abstract] OR "chronic pain"[Title/Abstract] OR "acute pain"[Title/Abstract] OR "overuse pain"[Title/Abstract] OR "chronic injury"[Title/Abstract] OR (("Chronic"[All Fields] OR "chronical"[All Fields] OR "chronically"[All Fields] OR "chronicities"[All Fields] OR "chronicity"[All Fields] OR "chronicization"[All Fields] OR "chronics"[All Fields]) AND "injuries knee"[Title/Abstract]) OR "nonspecific chronic pain"[Title/Abstract] OR "chronic widespread pain"[Title/Abstract] |  |  | (("Osteoarthritis"[Title/Abstract] OR "hip osteoarthritis"[Title/Abstract] OR "hand osteoarthritis"[Title/Abstract] OR "knee osteoarthritis"[Title/Abstract] OR "knee arthrosis"[Title/Abstract] OR "knee cartilage"[Title/Abstract] OR "degenerative arthritis"[Title/Abstract] OR "musculoskeletal pain"[Title/Abstract] OR "chronic pain"[Title/Abstract] OR "overuse pain"[Title/Abstract] OR "chronic injury"[Title/Abstract] OR (("Chronic"[All Fields] OR "chronical"[All Fields] OR "chronically"[All Fields] OR "chronicities"[All Fields] OR "chronicity"[All Fields] OR "chronicization"[All Fields] OR "chronics"[All Fields]) AND "injuries knee"[Title/Abstract]) OR "nonspecific chronic pain"[Title/Abstract] OR "chronic widespread pain"[Title/Abstract]) AND "Osteoarthritis"[Title/Abstract]) OR "knee osteoarthritis"[Title/Abstract] OR "knee arthrosis"[Title/Abstract] OR "knee cartilage"[Title/Abstract] OR "degenerative arthritis"[Title/Abstract] OR "musculoskeletal pain"[Title/Abstract] OR "chronic pain"[Title/Abstract] OR "acute pain"[Title/Abstract] OR "overuse pain"[Title/Abstract] OR "chronic injury"[Title/Abstract] OR (("Chronic"[All Fields] OR "chronical"[All Fields] OR "chronically"[All Fields] OR "chronicities"[All Fields] OR "chronicity"[All Fields] OR "chronicization"[All Fields] OR "chronics"[All Fields]) AND "injuries knee"[Title/Abstract]) OR "nonspecific chronic pain"[Title/Abstract] OR "chronic widespread pain"[Title/Abstract] | 182,083 | 6:03:39 | 2025/8/10 |
| 3 | ((((((((((double blind*[Title/Abstract]) OR (single blind*[Title/Abstract])) OR (placebo*[Title/Abstract])) OR (random*[Title/Abstract])) OR (prospective stud*[Title/Abstract])) OR (follow-up stud*[Title/Abstract])) OR (controlled trial*[Title/Abstract])) OR (evaluation stud*[Title/Abstract])) OR (comparative stud*[Title/Abstract])) OR (research design[Title/Abstract])) OR (clinical trial*[Title/Abstract]) |  |  | "double blind*"[Title/Abstract] OR "single blind*"[Title/Abstract] OR "placebo*"[Title/Abstract] OR "random*"[Title/Abstract] OR "prospective stud*"[Title/Abstract] OR "follow up stud*"[Title/Abstract] OR "controlled trial*"[Title/Abstract] OR "evaluation stud*"[Title/Abstract] OR "comparative stud*"[Title/Abstract] OR "research design"[Title/Abstract] OR "clinical trial*"[Title/Abstract] | 2,562,810 | 6:00:28 | 2025/8/10 |
| 2 | Virtual Medicine Medicine[Title/Abstract] OR Virtual Tele-Referral[Title/Abstract] OR Tele Referral[Title/Abstract] OR Tele-Referrals[Title/Abstract] OR Mobile Health[Title/Abstract] OR mHealth[Title/Abstract] OR Telehealth[Title/Abstract] OR eHealth[Title/Abstract] OR Tele-Intensive Care[Title/Abstract] OR Tele Intensive Care[Title/Abstract] OR Tele-ICU[Title/Abstract] OR Tele ICU[Title/Abstract] OR Telecare[Title/Abstract] OR Tele-Care[Title/Abstract] OR Tele Care[Title/Abstract] OR digital movement therapy[Title/Abstract] OR digital movement therapies[Title/Abstract] OR mobile health[Title/Abstract] OR etherapy[Title/Abstract] OR etherapies[Title/Abstract] OR web-based intervention[Title/Abstract] OR digital intervention[Title/Abstract] OR computer-based intervention[Title/Abstract] OR app-based intervention[Title/Abstract] OR digital health application[Title/Abstract] OR technology-assisted therapy[Title/Abstract] OR technology-assisted therapies[Title/Abstract] OR internet-based intervention[Title/Abstract] OR computer-assisted therapy[Title/Abstract] OR computer-assisted therapies[Title/Abstract] OR health app[Title/Abstract] OR mobile application[Title/Abstract] OR smartphone[Title/Abstract] OR telemedicine[Title/Abstract] OR online intervention[Title/Abstract] OR internet-delivered intervention[Title/Abstract] OR mobile application[Title/Abstract] OR smartphone application[Title/Abstract] OR sensors[Title/Abstract] OR connected watch[Title/Abstract] OR Telemedicine[Title/Abstract] OR Telerehabilitation[Title/Abstract] OR Internet[Title/Abstract] OR Internet-Based Intervention[Title/Abstract] OR Online[Title/Abstract] OR App[Title/Abstract] OR m-health[Title/Abstract] OR smartphone[Title/Abstract] OR mobile*[Title/Abstract] OR iphone[Title/Abstract] OR ipad[Title/Abstract] OR app[Title/Abstract] OR apps[Title/Abstract] OR application*[Title/Abstract] OR telemedicine[Title/Abstract] OR telerehabilitation[Title/Abstract] OR internet rehabilitation[Title/Abstract] OR web[Title/Abstract] OR online[Title/Abstract] OR app[Title/Abstract] OR wearable[Title/Abstract] OR sensor[Title/Abstract] OR mobile applications[Title/Abstract] OR telemedicine[Title/Abstract] OR Internet-based[Title/Abstract] OR web-based[Title/Abstract] OR APP∗[Title/Abstract] |  |  | (("virtual"[All Fields] OR "virtuality"[All Fields] OR "virtualization"[All Fields] OR "virtualized"[All Fields] OR "virtualizing"[All Fields] OR "virtuals"[All Fields]) AND "medicine medicine"[Title/Abstract]) OR (("virtual"[All Fields] OR "virtuality"[All Fields] OR "virtualization"[All Fields] OR "virtualized"[All Fields] OR "virtualizing"[All Fields] OR "virtuals"[All Fields]) AND "Tele-Referral"[Title/Abstract]) OR "Tele-Referral"[Title/Abstract] OR "Tele-Referrals"[Title/Abstract] OR "mobile health"[Title/Abstract] OR "mHealth"[Title/Abstract] OR "Telehealth"[Title/Abstract] OR "eHealth"[Title/Abstract] OR "tele intensive care"[Title/Abstract] OR "tele intensive care"[Title/Abstract] OR "Tele-ICU"[Title/Abstract] OR "Tele-ICU"[Title/Abstract] OR "Telecare"[Title/Abstract] OR "Tele-Care"[Title/Abstract] OR "Tele-Care"[Title/Abstract] OR (("digitalisation"[All Fields] OR "digitalised"[All Fields] OR "digitalization"[All Fields] OR "digitalize"[All Fields] OR "digitalized"[All Fields] OR "digitalizer"[All Fields] OR "digitalizing"[All Fields] OR "digitally"[All Fields] OR "digitals"[All Fields] OR "digitization"[All Fields] OR "digitizations"[All Fields] OR "digitize"[All Fields] OR "digitized"[All Fields] OR "digitizer"[All Fields] OR "digitizers"[All Fields] OR "digitizes"[All Fields] OR "digitizing"[All Fields] OR "radiographic image enhancement"[MeSH Terms] OR ("radiographic"[All Fields] AND "image"[All Fields] AND "enhancement"[All Fields]) OR "radiographic image enhancement"[All Fields] OR "digital"[All Fields]) AND "movement therapy"[Title/Abstract]) OR (("digitalisation"[All Fields] OR "digitalised"[All Fields] OR "digitalization"[All Fields] OR "digitalize"[All Fields] OR "digitalized"[All Fields] OR "digitalizer"[All Fields] OR "digitalizing"[All Fields] OR "digitally"[All Fields] OR "digitals"[All Fields] OR "digitization"[All Fields] OR "digitizations"[All Fields] OR "digitize"[All Fields] OR "digitized"[All Fields] OR "digitizer"[All Fields] OR "digitizers"[All Fields] OR "digitizes"[All Fields] OR "digitizing"[All Fields] OR "radiographic image enhancement"[MeSH Terms] OR ("radiographic"[All Fields] AND "image"[All Fields] AND "enhancement"[All Fields]) OR "radiographic image enhancement"[All Fields] OR "digital"[All Fields]) AND "movement therapies"[Title/Abstract]) OR "mobile health"[Title/Abstract] OR "etherapy"[Title/Abstract] OR "etherapies"[Title/Abstract] OR "web based intervention"[Title/Abstract] OR "digital intervention"[Title/Abstract] OR "computer based intervention"[Title/Abstract] OR "app based intervention"[Title/Abstract] OR "digital health application"[Title/Abstract] OR "technology assisted therapy"[Title/Abstract] OR "technology assisted therapies"[Title/Abstract] OR "internet based intervention"[Title/Abstract] OR "computer assisted therapy"[Title/Abstract] OR "computer assisted therapies"[Title/Abstract] OR "health app"[Title/Abstract] OR "mobile application"[Title/Abstract] OR "smartphone"[Title/Abstract] OR "Telemedicine"[Title/Abstract] OR "online intervention"[Title/Abstract] OR "internet delivered intervention"[Title/Abstract] OR "mobile application"[Title/Abstract] OR "smartphone application"[Title/Abstract] OR "sensors"[Title/Abstract] OR "connected watch"[Title/Abstract] OR "Telemedicine"[Title/Abstract] OR "Telerehabilitation"[Title/Abstract] OR "Internet"[Title/Abstract] OR "internet based intervention"[Title/Abstract] OR "Online"[Title/Abstract] OR "app"[Title/Abstract] OR "m-health"[Title/Abstract] OR "smartphone"[Title/Abstract] OR "mobile*"[Title/Abstract] OR "iphone"[Title/Abstract] OR "ipad"[Title/Abstract] OR "app"[Title/Abstract] OR "apps"[Title/Abstract] OR "application*"[Title/Abstract] OR "Telemedicine"[Title/Abstract] OR "Telerehabilitation"[Title/Abstract] OR "internet rehabilitation"[Title/Abstract] OR "web"[Title/Abstract] OR "Online"[Title/Abstract] OR "app"[Title/Abstract] OR "wearable"[Title/Abstract] OR "sensor"[Title/Abstract] OR "mobile applications"[Title/Abstract] OR "Telemedicine"[Title/Abstract] OR "internet based"[Title/Abstract] OR "web-based"[Title/Abstract] OR "app"[Title/Abstract] | 2,836,611 | 5:59:54 | 2025/8/10 |
| 1 | movement therapy[Title/Abstract] OR movement therapies[Title/Abstract] OR physical therapy[Title/Abstract] OR physical therapies[Title/Abstract] OR therapeutic exercise[Title/Abstract] OR medical gymnastic[Title/Abstract] OR traditional therapy[Title/Abstract] OR traditional therapies[Title/Abstract] OR manual therapy[Title/Abstract] OR manual therapies[Title/Abstract] OR physiotherapy[Title/Abstract] OR exercis*[Title/Abstract] OR exertion*[Title/Abstract] OR training*[Title/Abstract] OR sport[Title/Abstract] OR strength*[Title/Abstract] OR isometric*[Title/Abstract] OR isotonic*[Title/Abstract] OR isokinetic*[Title/Abstract] OR aerobic*[Title/Abstract] OR endurance[Title/Abstract] OR weight*[Title/Abstract] OR exercis*[Title/Abstract] OR train*[Title/Abstract] OR muscle strength*[Title/Abstract] OR physical endurance[Title/Abstract] OR physiotherap*[Title/Abstract] OR therapy[Title/Abstract] OR therapies[Title/Abstract] OR rehabilitation[Title/Abstract] OR physical activity[Title/Abstract] OR physical exercise[Title/Abstract] OR exercises[Title/Abstract] OR physical activity[Title/Abstract] OR activities, physical[Title/Abstract] OR activity, physical[Title/Abstract] OR physical activities[Title/Abstract] OR exercise, physical[Title/Abstract] OR exercises, physical[Title/Abstract] OR physical exercise[Title/Abstract] OR physical exercises[Title/Abstract] OR acute exercise[Title/Abstract] OR acute exercises[Title/Abstract] OR exercise, acute[Title/Abstract] OR exercises, acute[Title/Abstract] OR exercise, isometric[Title/Abstract] OR exercises, isometric[Title/Abstract] OR isometric exercises[Title/Abstract] OR isometric exercise[Title/Abstract] OR exercise, aerobic[Title/Abstract] OR aerobic exercise[Title/Abstract] OR aerobic exercises[Title/Abstract] OR exercises, aerobic[Title/Abstract] OR exercise training[Title/Abstract] OR exercise trainings[Title/Abstract] OR training, exercise[Title/Abstract] OR trainings, exercise[Title/Abstract] OR football:ti,ab,kw[Title/Abstract] OR tai chi[Title/Abstract] OR basketball:ti,ab,kw[Title/Abstract] OR high-intensity interval training[Title/Abstract] OR high intensity interval training[Title/Abstract] OR high-intensity interval trainings[Title/Abstract] OR interval training, high-intensity[Title/Abstract] OR interval trainings, high-intensity[Title/Abstract] OR training, high-intensity interval[Title/Abstract] OR trainings, high-intensity interval[Title/Abstract] OR high-intensity intermittent exercise[Title/Abstract] OR exercise, high-intensity intermittent[Title/Abstract] OR exercises, high-intensity intermittent[Title/Abstract] OR high-intensity intermittent exercises[Title/Abstract] OR sprint interval training[Title/Abstract] OR sprint interval trainings[Title/Abstract] |  |  | "movement therapy"[Title/Abstract] OR "movement therapies"[Title/Abstract] OR "physical therapy"[Title/Abstract] OR "physical therapies"[Title/Abstract] OR "therapeutic exercise"[Title/Abstract] OR "medical gymnastic"[Title/Abstract] OR "traditional therapy"[Title/Abstract] OR "traditional therapies"[Title/Abstract] OR "manual therapy"[Title/Abstract] OR "manual therapies"[Title/Abstract] OR "physiotherapy"[Title/Abstract] OR "exercis*"[Title/Abstract] OR "exertion*"[Title/Abstract] OR "training*"[Title/Abstract] OR "sport"[Title/Abstract] OR "strength*"[Title/Abstract] OR "isometric*"[Title/Abstract] OR "isotonic*"[Title/Abstract] OR "isokinetic*"[Title/Abstract] OR "aerobic*"[Title/Abstract] OR "endurance"[Title/Abstract] OR "weight*"[Title/Abstract] OR "exercis*"[Title/Abstract] OR "train*"[Title/Abstract] OR "muscle strength*"[Title/Abstract] OR "physical endurance"[Title/Abstract] OR "physiotherap*"[Title/Abstract] OR "therapy"[Title/Abstract] OR "therapies"[Title/Abstract] OR "rehabilitation"[Title/Abstract] OR "physical activity"[Title/Abstract] OR "physical exercise"[Title/Abstract] OR "exercises"[Title/Abstract] OR "physical activity"[Title/Abstract] OR "activities physical"[Title/Abstract] OR "activity physical"[Title/Abstract] OR "physical activities"[Title/Abstract] OR "exercise physical"[Title/Abstract] OR "exercises physical"[Title/Abstract] OR "physical exercise"[Title/Abstract] OR "physical exercises"[Title/Abstract] OR "acute exercise"[Title/Abstract] OR "acute exercises"[Title/Abstract] OR "exercise acute"[Title/Abstract] OR "exercises acute"[Title/Abstract] OR "exercise isometric"[Title/Abstract] OR "exercises isometric"[Title/Abstract] OR "isometric exercises"[Title/Abstract] OR "isometric exercise"[Title/Abstract] OR "exercise aerobic"[Title/Abstract] OR "aerobic exercise"[Title/Abstract] OR "aerobic exercises"[Title/Abstract] OR "exercises aerobic"[Title/Abstract] OR "exercise training"[Title/Abstract] OR "exercise trainings"[Title/Abstract] OR "training exercise"[Title/Abstract] OR (("education"[MeSH Subheading] OR "education"[All Fields] OR "training"[All Fields] OR "education"[MeSH Terms] OR "train"[All Fields] OR "train s"[All Fields] OR "trained"[All Fields] OR "training s"[All Fields] OR "trainings"[All Fields] OR "trains"[All Fields]) AND "exercise"[Title/Abstract]) OR ((("football"[MeSH Terms] OR "football"[All Fields] OR "football s"[All Fields] OR "footballer"[All Fields] OR "footballer s"[All Fields] OR "footballers"[All Fields] OR "footballs"[All Fields]) AND "ti ab"[All Fields]) AND "kw"[Title/Abstract]) OR "tai chi"[Title/Abstract] OR ((("basketball"[MeSH Terms] OR "basketball"[All Fields] OR "basketballs"[All Fields] OR "basketballers"[All Fields]) AND "ti ab"[All Fields]) AND "kw"[Title/Abstract]) OR "high intensity interval training"[Title/Abstract] OR "high intensity interval training"[Title/Abstract] OR "high intensity interval trainings"[Title/Abstract] OR "interval training high intensity"[Title/Abstract] OR ((("interval"[All Fields] OR "intervals"[All Fields]) AND ("education"[MeSH Subheading] OR "education"[All Fields] OR "training"[All Fields] OR "education"[MeSH Terms] OR "train"[All Fields] OR "train s"[All Fields] OR "trained"[All Fields] OR "training s"[All Fields] OR "trainings"[All Fields] OR "trains"[All Fields])) AND "high-intensity"[Title/Abstract]) OR "training high intensity interval"[Title/Abstract] OR (("education"[MeSH Subheading] OR "education"[All Fields] OR "training"[All Fields] OR "education"[MeSH Terms] OR "train"[All Fields] OR "train s"[All Fields] OR "trained"[All Fields] OR "training s"[All Fields] OR "trainings"[All Fields] OR "trains"[All Fields]) AND "high intensity interval"[Title/Abstract]) OR "high intensity intermittent exercise"[Title/Abstract] OR "exercise high intensity intermittent"[Title/Abstract] OR (("exercise"[MeSH Terms] OR "exercise"[All Fields] OR "exercises"[All Fields] OR "exercise therapy"[MeSH Terms] OR ("exercise"[All Fields] AND "therapy"[All Fields]) OR "exercise therapy"[All Fields] OR "exercising"[All Fields] OR "exercise s"[All Fields] OR "exercised"[All Fields] OR "exerciser"[All Fields] OR "exercisers"[All Fields]) AND "high intensity intermittent"[Title/Abstract]) OR "high intensity intermittent exercises"[Title/Abstract] OR "sprint interval training"[Title/Abstract] OR "sprint interval trainings"[Title/Abstract] | 6,090,142 | 5:59:23 | 2025/8/10 |

**Web of science**

| Entitlements | Type | Search Query | Database | Results | Date Run |
| --- | --- | --- | --- | --- | --- |
| - WOS: 1985 to 2025 - CSCD: 1989 to 2025 - KJD: 1980 to 2025 - MEDLINE: 1950 to 2025 - PPRN: 1991 to 2025 - PQDT: 1637 to 2025 - SCIELO: 2002 to 2025 | Search | double blind* OR single blind* OR placebo* OR random* OR prospective stud* OR follow-up stud* OR controlled trial* OR evaluation stud* OR comparative stud* OR research design OR clinical trial* (Topic) AND Aged OR elderly OR older people OR older adults OR aged patient OR aged people OR aged person OR aged subject OR elderly patient OR elderly people OR elderly person OR elderly subject OR senior citizen OR senium (Topic) AND movement therapy OR movement therapies OR physical therapy OR physical therapies OR therapeutic exercise OR medical gymnastic OR traditional therapy OR traditional therapies OR manual therapy OR manual therapies OR physiotherapy OR exercis* OR exertion* OR training* OR sport OR strength* OR isometric* OR isotonic* OR isokinetic* OR aerobic* OR endurance OR weight* OR train* OR muscle strength* OR physical endurance OR physiotherap* OR therapy OR therapies OR rehabilitation OR physical activity OR physical exercise OR exercises OR activities, physical OR activity, physical OR physical activities OR exercise, physical OR exercises, physical OR physical exercises OR acute exercise OR acute exercises OR exercise, acute OR exercises, acute OR exercise, isometric OR exercises, isometric OR isometric exercises OR isometric exercise OR exercise, aerobic OR aerobic exercise OR aerobic exercises OR exercises, aerobic OR exercise training OR exercise trainings OR training, exercise OR trainings, exercise OR football OR tai chi OR basketball OR high-intensity interval training OR high intensity interval training OR high-intensity interval trainings OR interval training, high-intensity OR interval trainings, high-intensity OR training, high-intensity interval OR trainings, high-intensity interval OR high-intensity intermittent exercise OR exercise, high-intensity intermittent OR exercises, high-intensity intermittent OR high-intensity intermittent exercises OR sprint interval training OR sprint interval trainings (Topic) AND Telemedicine OR mHealth OR Telehealth OR eHealth OR Tele-Intensive Care OR Telecare OR Tele-Care OR digital movement therapy OR digital movement therapies OR mobile health OR etherapy OR etherapies OR web-based intervention OR digital intervention OR computer-based intervention OR app-based intervention OR digital health application OR technology-assisted therapy OR technology-assisted therapies OR internet-based intervention OR computer-assisted therapy OR computer-assisted therapies OR health app OR mobile application OR smartphone OR online intervention OR internet-delivered intervention OR smartphone application OR sensors OR connected watch OR Telerehabilitation OR Internet OR Internet-Based Intervention OR Online OR App OR m-health OR mobile* OR iphone OR ipad OR apps OR application* OR internet rehabilitation OR web OR wearable OR mobile applications OR Internet-based OR web-based OR APP∗ (Topic) AND Osteoarthritis OR knee Osteoarthritis OR hip osteoarthritis OR knee arthrosis OR degenerative arthritis OR musculoskeletal pain OR chronic pain OR acute pain OR overuse pain OR chronic injury OR chronic injuries knee OR Nonspecific chronic pain OR Chronic widespread pain (Topic) and Preprint Citation Index (Exclude – Database) | All Databases | 14973 | Sun Aug 10 2025 18:54:34 GMT+0800 |
