# Supplementary figures and images for: Effectiveness of telemedicine-delivered exercise interventions in older adult patients with osteoarthritis: a systematic review and meta-analysis
Source: Front Public Health. 2025 Dec 3;13:1719841. doi: 10.3389/fpubh.2025.1719841 (PMC12708276; doi:10.3389/fpubh.2025.1719841)

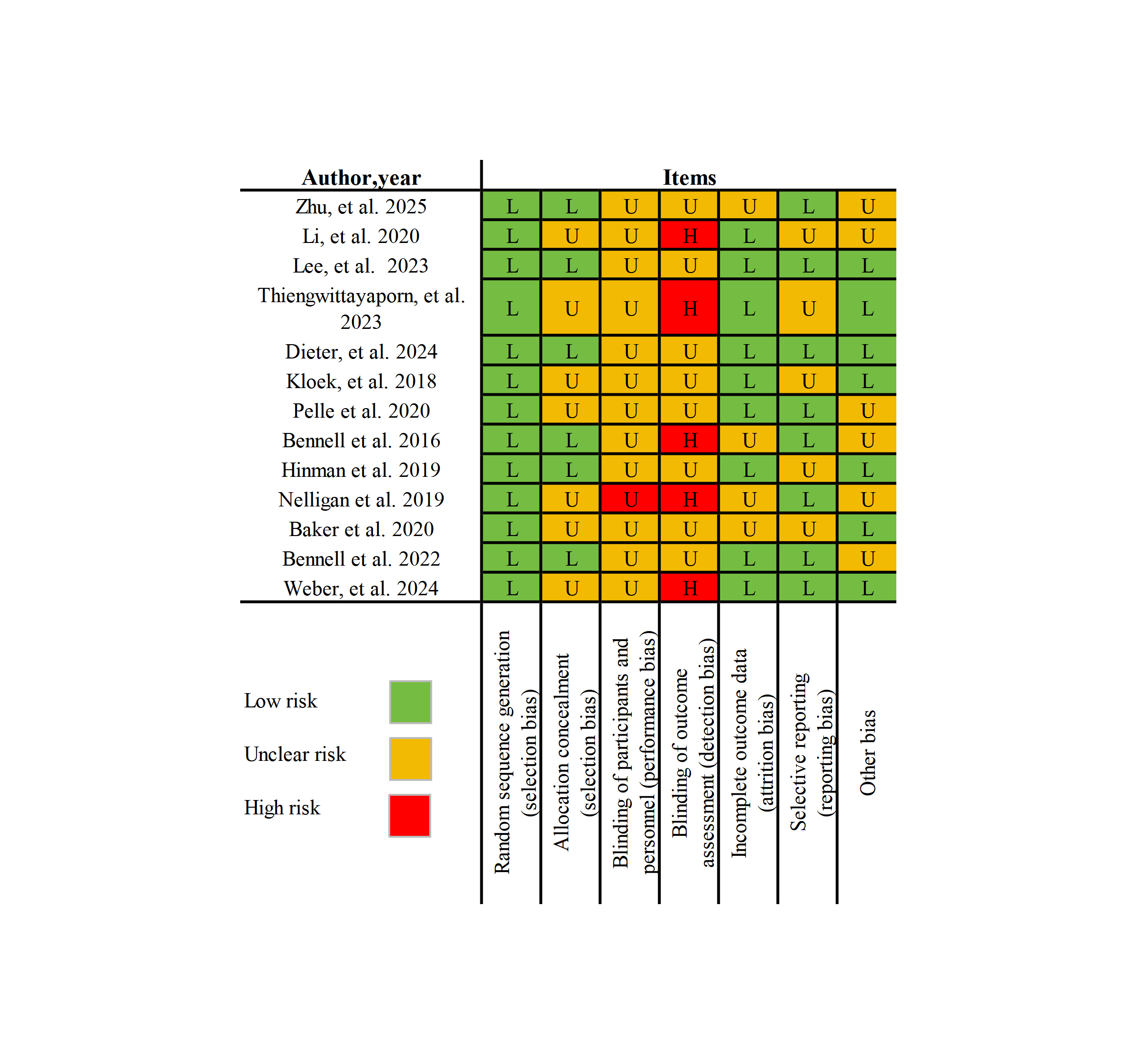

Supplement: Supplementary file 2 [file Image_1.TIF]

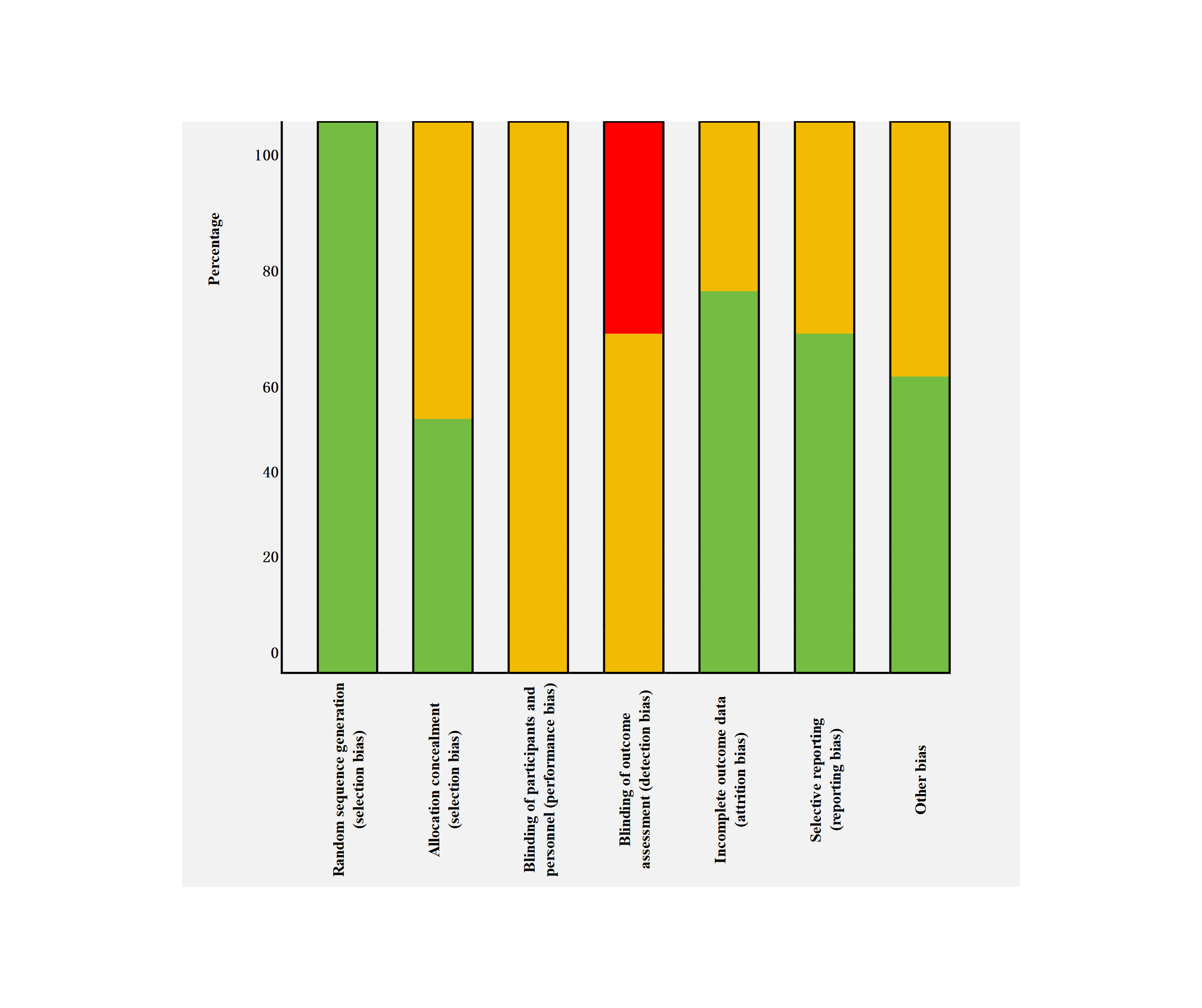

Supplement: Supplementary file 3 [file Image_2.TIF]

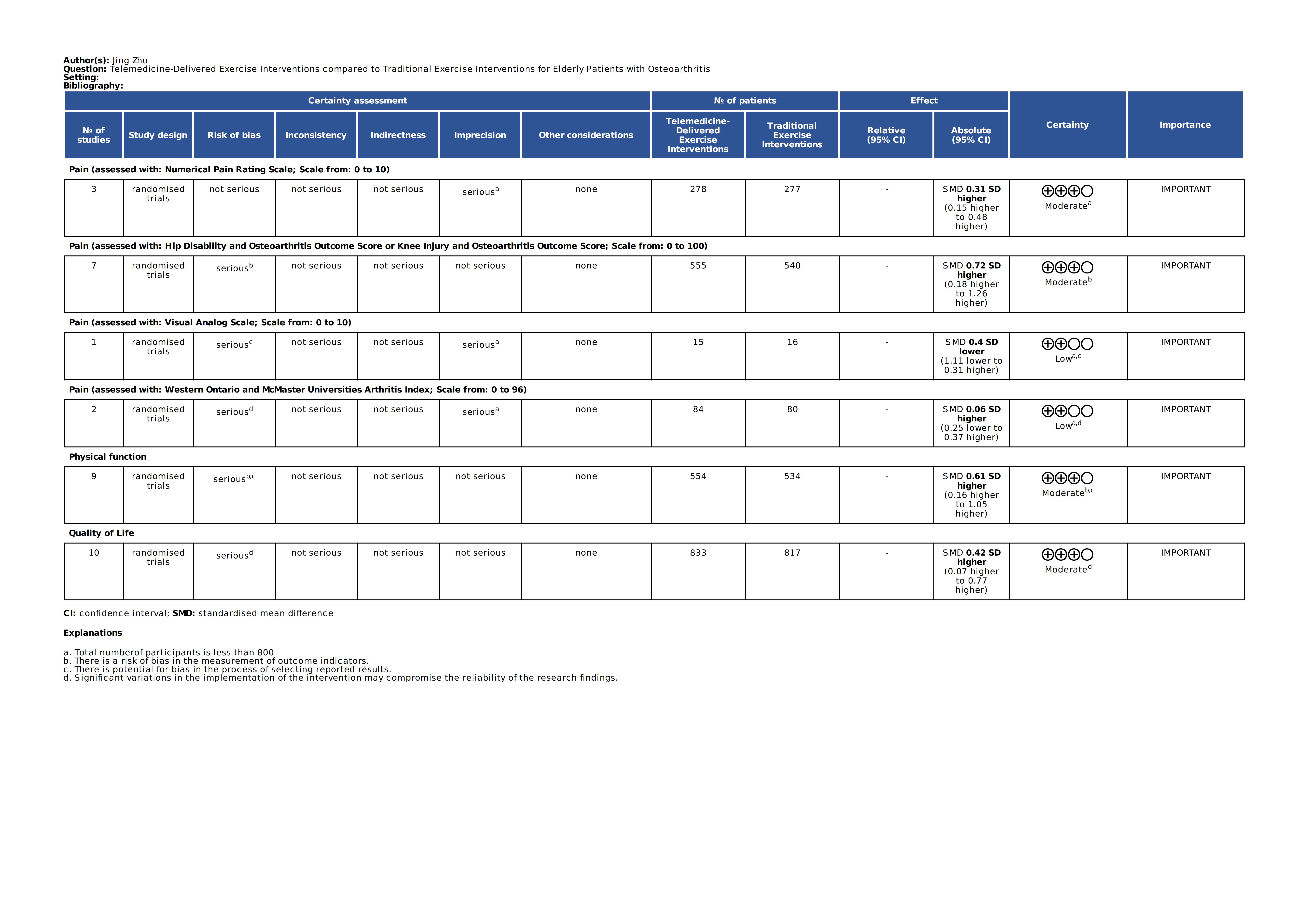

Supplement: Supplementary file 4 [file Image_3.JPEG]

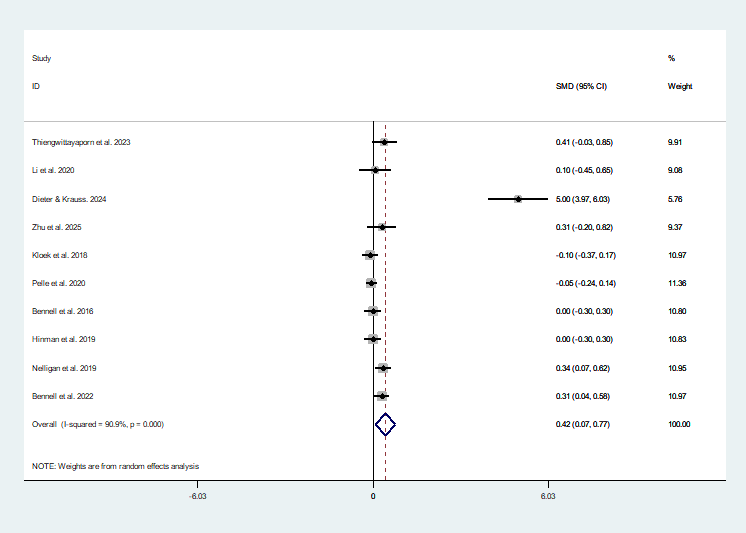

Supplement: Supplementary file 5 [file Image_4.TIF]

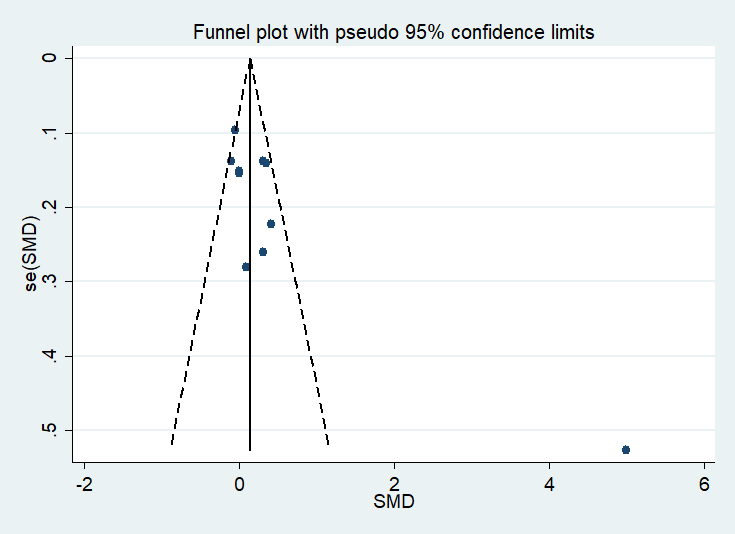

Supplement: Supplementary file 6 [file Image_5.TIF]
